# Supplementary material for: Tracking HIV-1 recombination to resolve its contribution to HIV-1 evolution in natural infection
Source: Nat Commun. 2018 May 15;9:1928. doi: 10.1038/s41467-018-04217-5 (PMC5954121; doi:10.1038/s41467-018-04217-5)
Supplement: Supplementary file 1 — Supplementary Information [file 41467_2018_4217_MOESM1_ESM.pdf]

# Supplementary Information

## Tracking HIV-1 recombination to resolve its contribution to HIV-1 evolution in natural infection

Song et al.

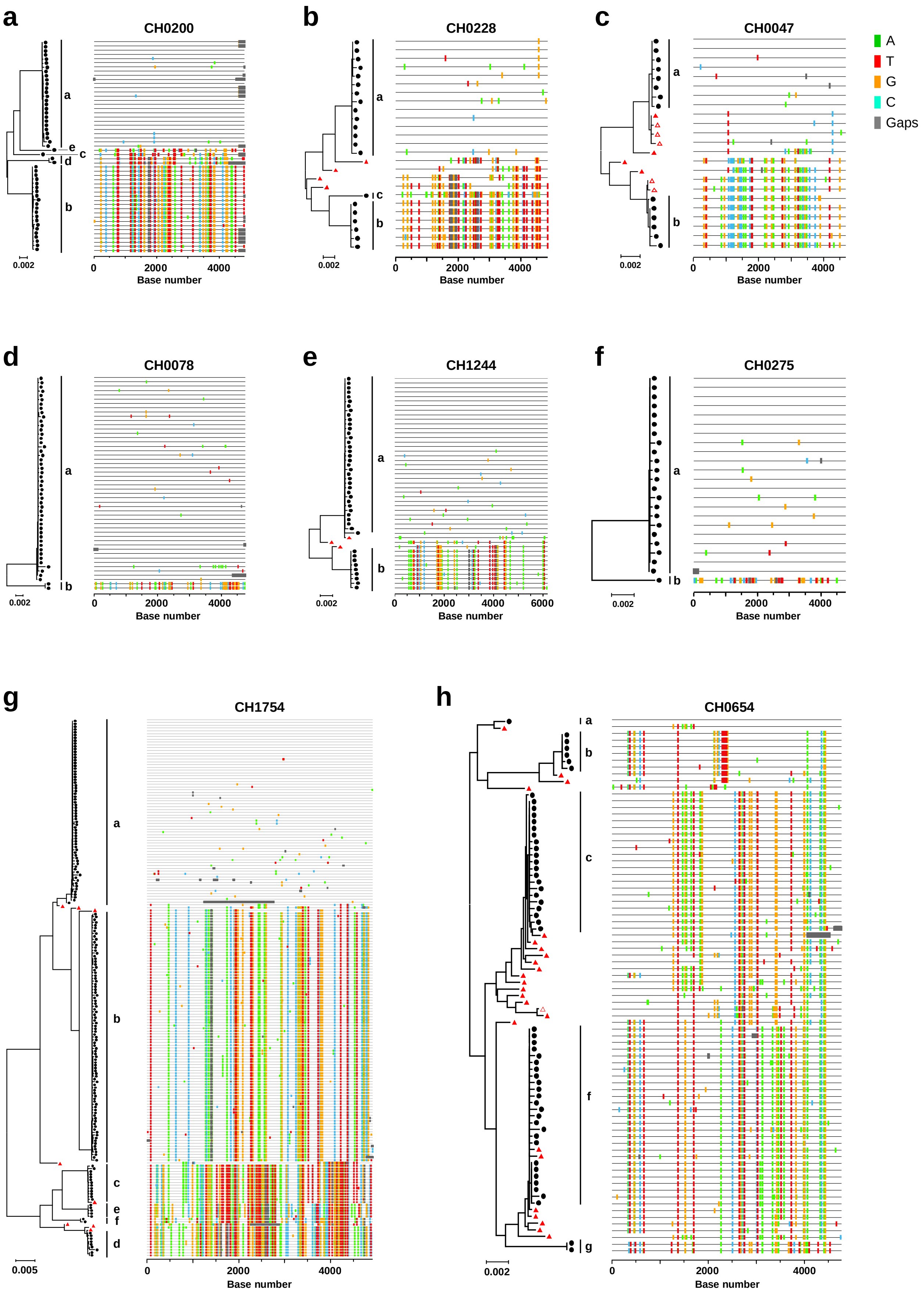

a
CH0200
b
CH0228
c
CH0047
A
T
G
C
Gaps
d
CH0078
e
CH1244
f
CH0275
g
CH1754
h
CH0654
0.002
0.005
0
2000
4000
6000
Base number

**Supplementary Figure 1**. **Determination of T/F and recombinant viruses within the 3' half genome sequences at screening.** T/F viruses were determined from screening sample sequences using phylogenetic trees and Highlighter plots. A T/F virus is defined as the consensus sequence representing each lineage of viruses from the screening time point that was too distinctive to have evolved through *de novo* post-transmission mutations. Here we show the 3' half genomes for subjects CH0200 **(a)**, CH0228 **(b)**, CH0047 **(c)**, CH0078 **(d)**, CH1244 **(e)**, CH0275 **(f)**, CH1754 **(g)** and CH0654 **(h)**. T/F viruses and their lineages are shown as black leaves on the trees. T/F lineages are labeled with lowercase letters, and recombinant sequences are shown as red triangles on the phylogenetic trees, with solid triangles for *de novo* recombinants and open triangles for descendants of recombinants. Phylogenetic trees were constructed using neighbor-joining methods and the Kimura 2-parameter model for mutations. Highlighter plots show nucleotide substitutions compared to the major T/F in each subject (first sequence at the top), and color-coded nucleotide substitutions.

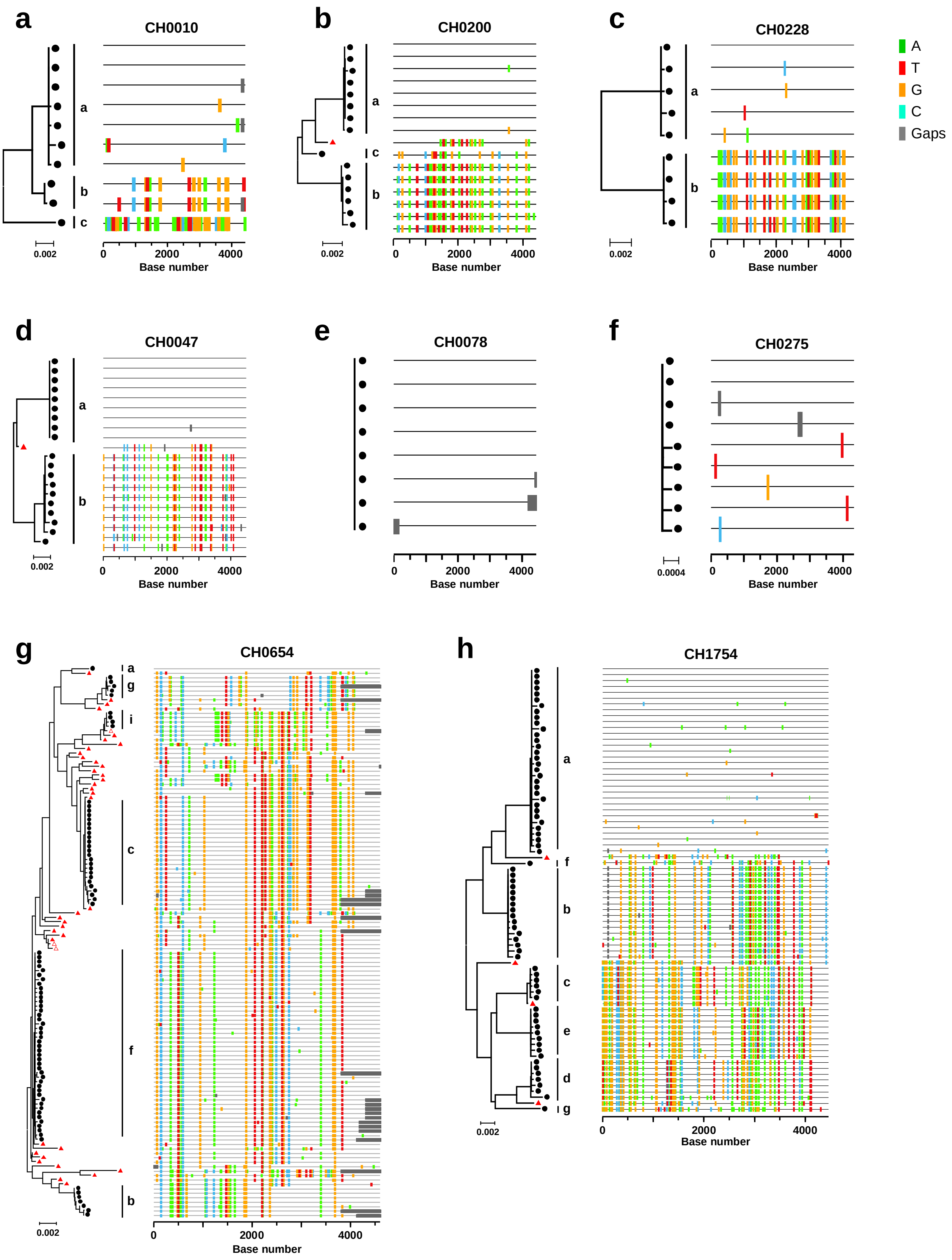
a
CH0010
a
b
c
0.002
0
2000
4000
Base number
b
CH0200
a
c
b
0.002
c
CH0228
a
b
0.002
A
T
G
C
Gaps
d
CH0047
a
b
0.002
e
CH0078
f
CH0275
0.0004
g
CH0654
a
g
i
c
f
b
0.002
h
CH1754
a
f
b
c
e
d
g
0.002
Base number

**Supplementary Figure 2**. **Determination of T/F and recombinant viruses within the 5' half genome sequences at screening**. Phylogenetic trees and Highlighter plots of the 5' half genome sequences obtained at screening for subjects CH0010 **(a)**, CH0200 **(b)**, CH0228 **(c)**, CH0047 **(d)**, CH0078 **(e)**, CH0275 **(f)**, CH1754 **(g)** and CH0654 **(h)**. T/F viruses are shown as black leaves on the trees. T/F lineages are labeled using lowercase letters, and recombinant sequences are shown as red triangles in the phylogenetic trees, with solid triangles for *de novo* recombinants and open triangles for descendants of particular recombinants. All phylogenetic trees were constructed using the neighbor-joining method and Kimura 2-parameter model for mutations. Highlighter plots show nucleotide substitutions compared to the major T/F in each subject (first sequence at the top), and color-coded nucleotide substitutions.

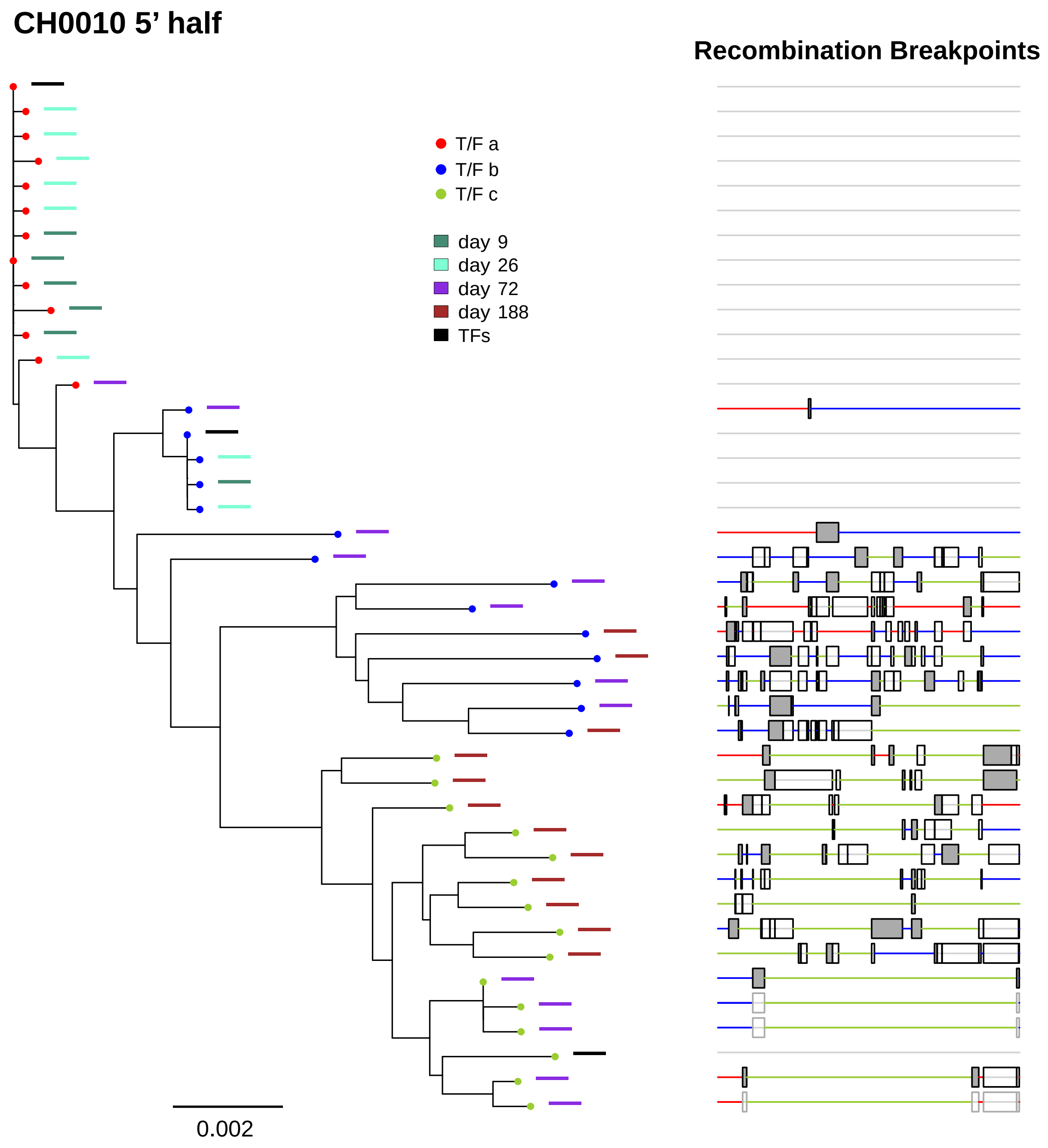


**Supplementary Figure 3. RAPR output figure for CH0010 5' half genome.**

**a**

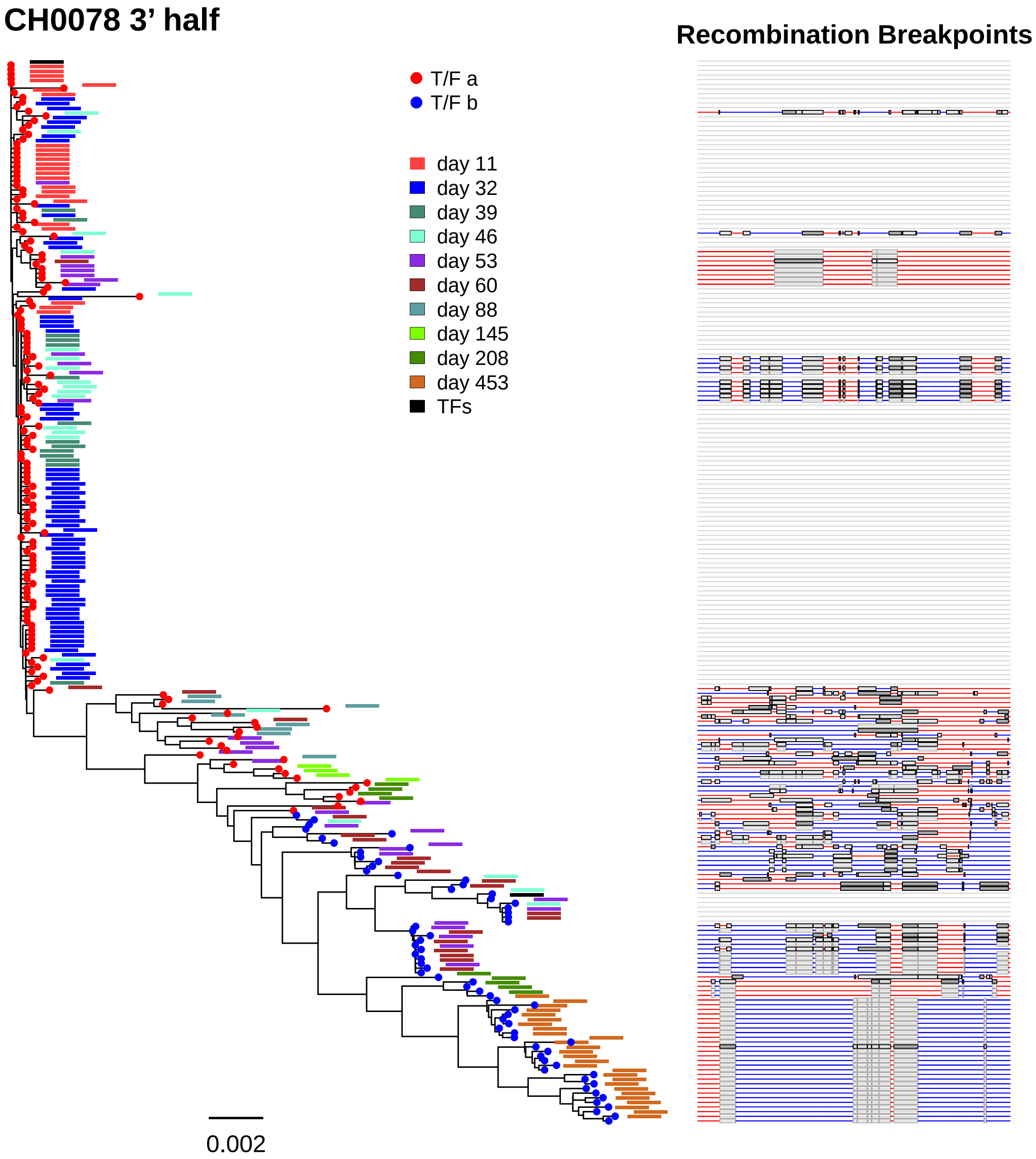
CH0078 3' half
Recombination Breakpoints
T/F a
T/F b
day 11
day 32
day 39
day 46
day 53
day 60
day 88
day 145
day 208
day 453
TFs
0.002

**b**

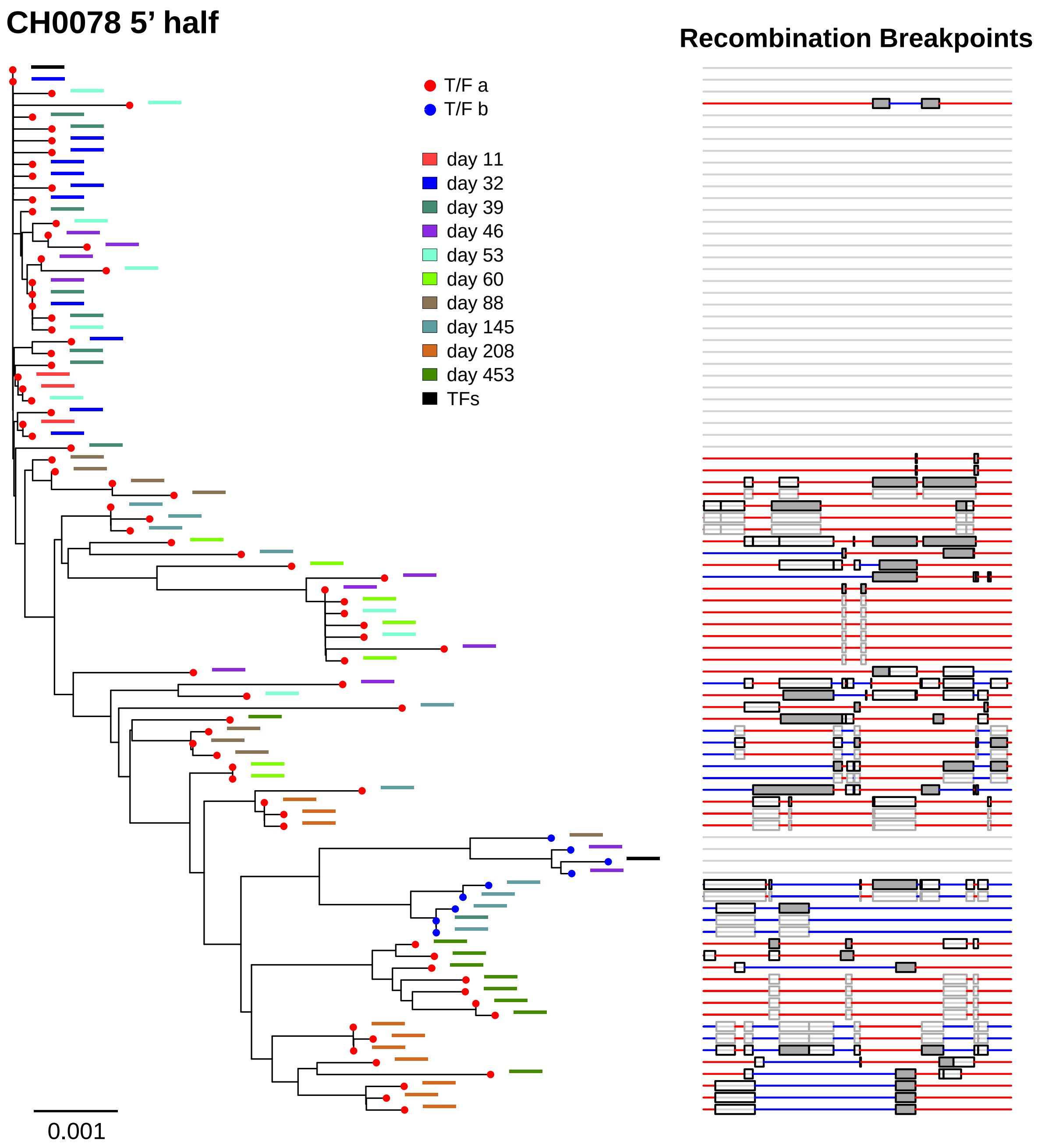


**Supplementary Figure 4. RAPR output figures for CH0078 3' half genome (a) and 5' half genome (b).**

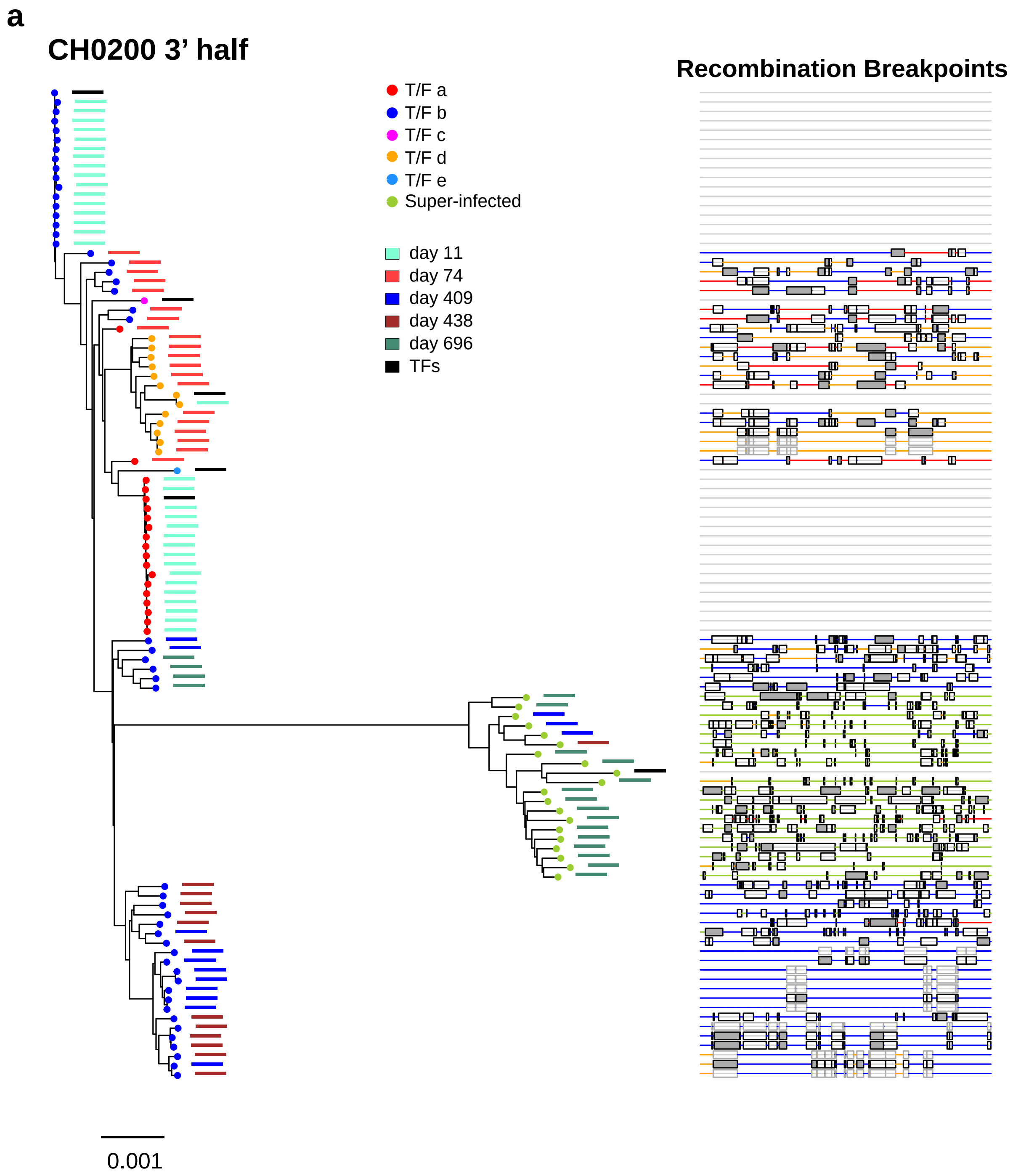
a
CH0200 3’ half
Recombination Breakpoints
T/F a
T/F b
T/F c
T/F d
T/F e
Super-infected
day 11
day 74
day 409
day 438
day 696
TFs
0.001

**b**

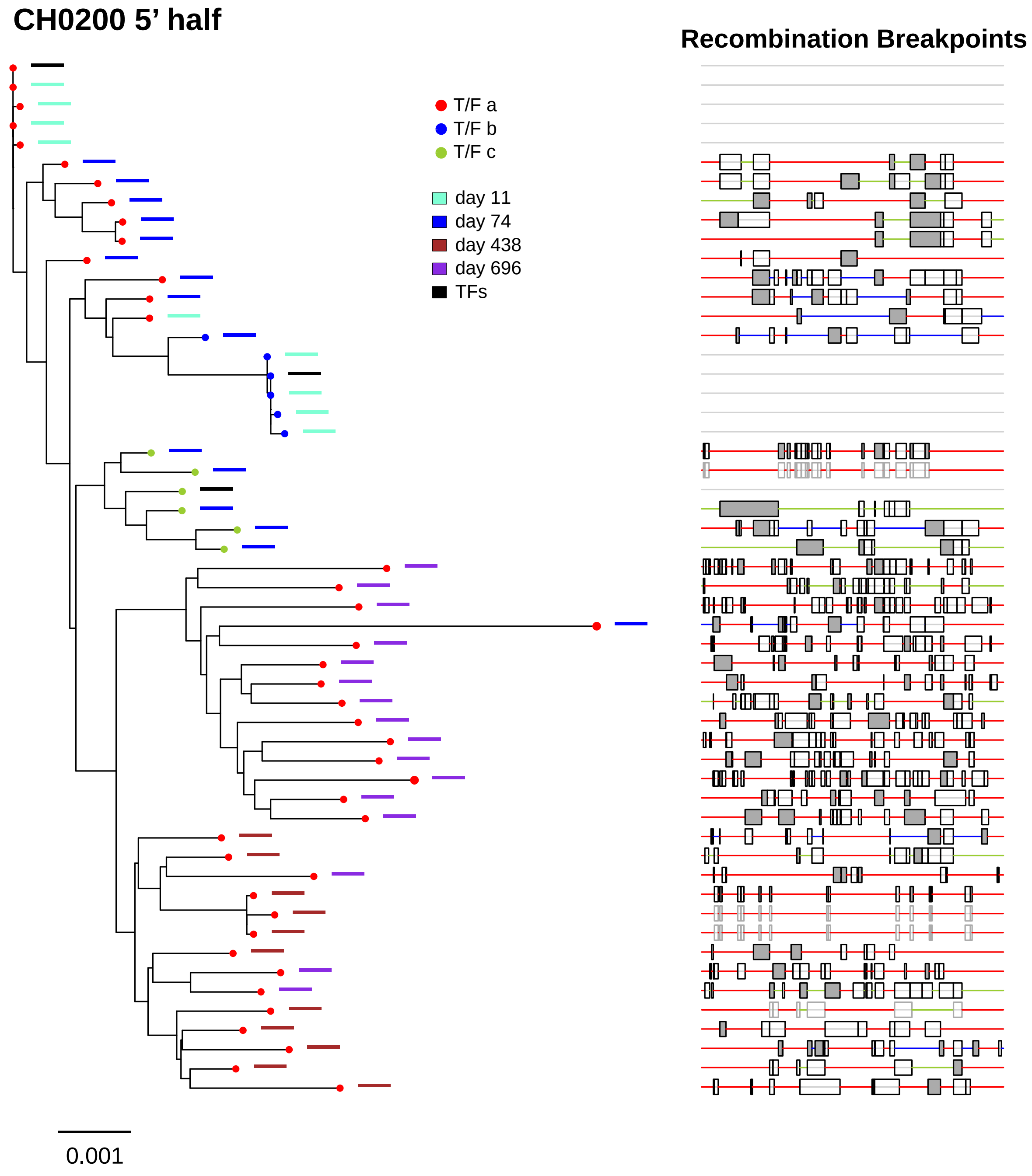


**Supplementary Figure 5. RAPR output figures for CH0200 3' half genome (a) and 5' half genome (b).**

**a**

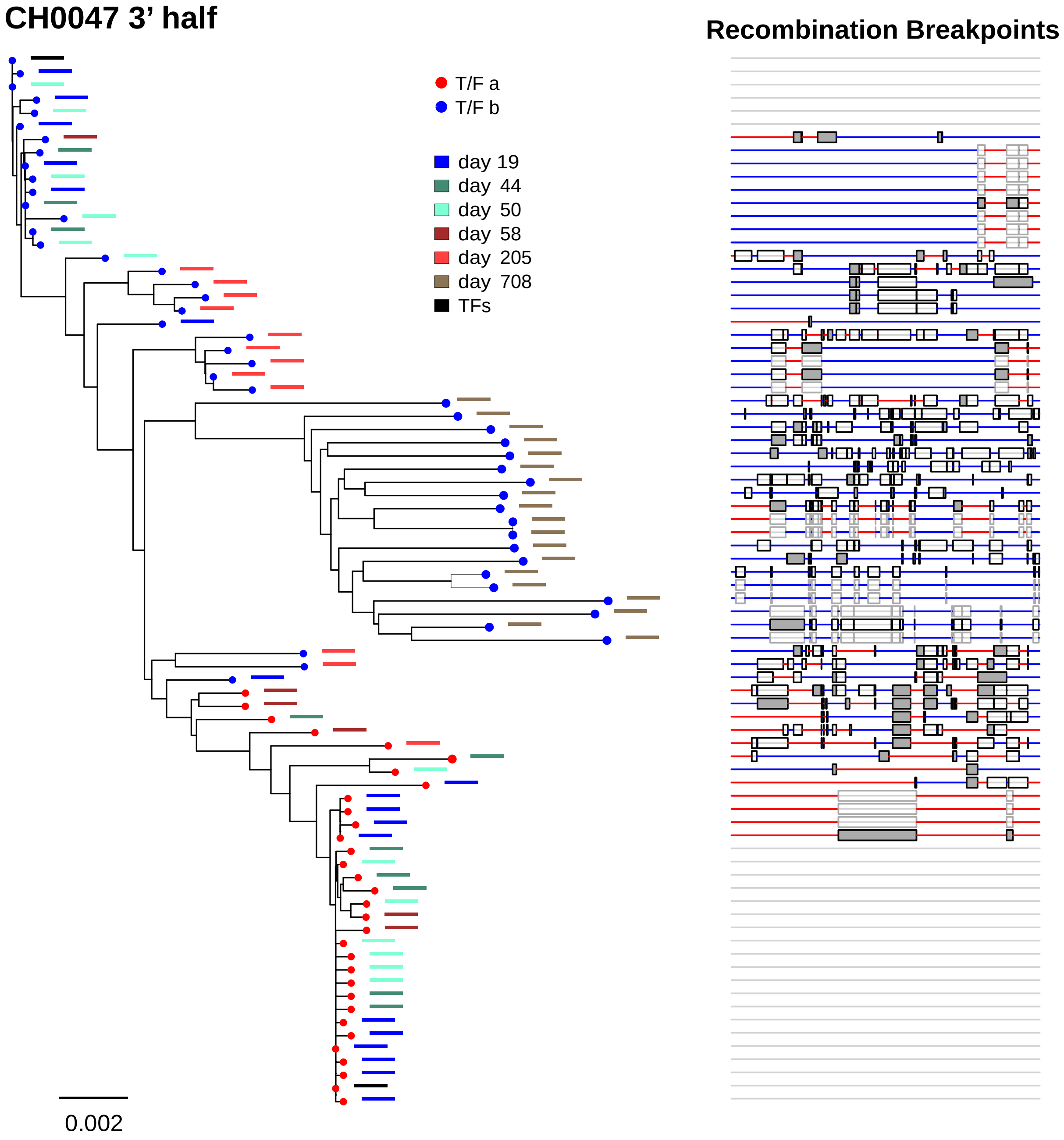

CH0047 3' half
T/F a
T/F b
day 19
day 44
day 50
day 58
day 205
day 708
TFs
0.002
Recombination Breakpoints

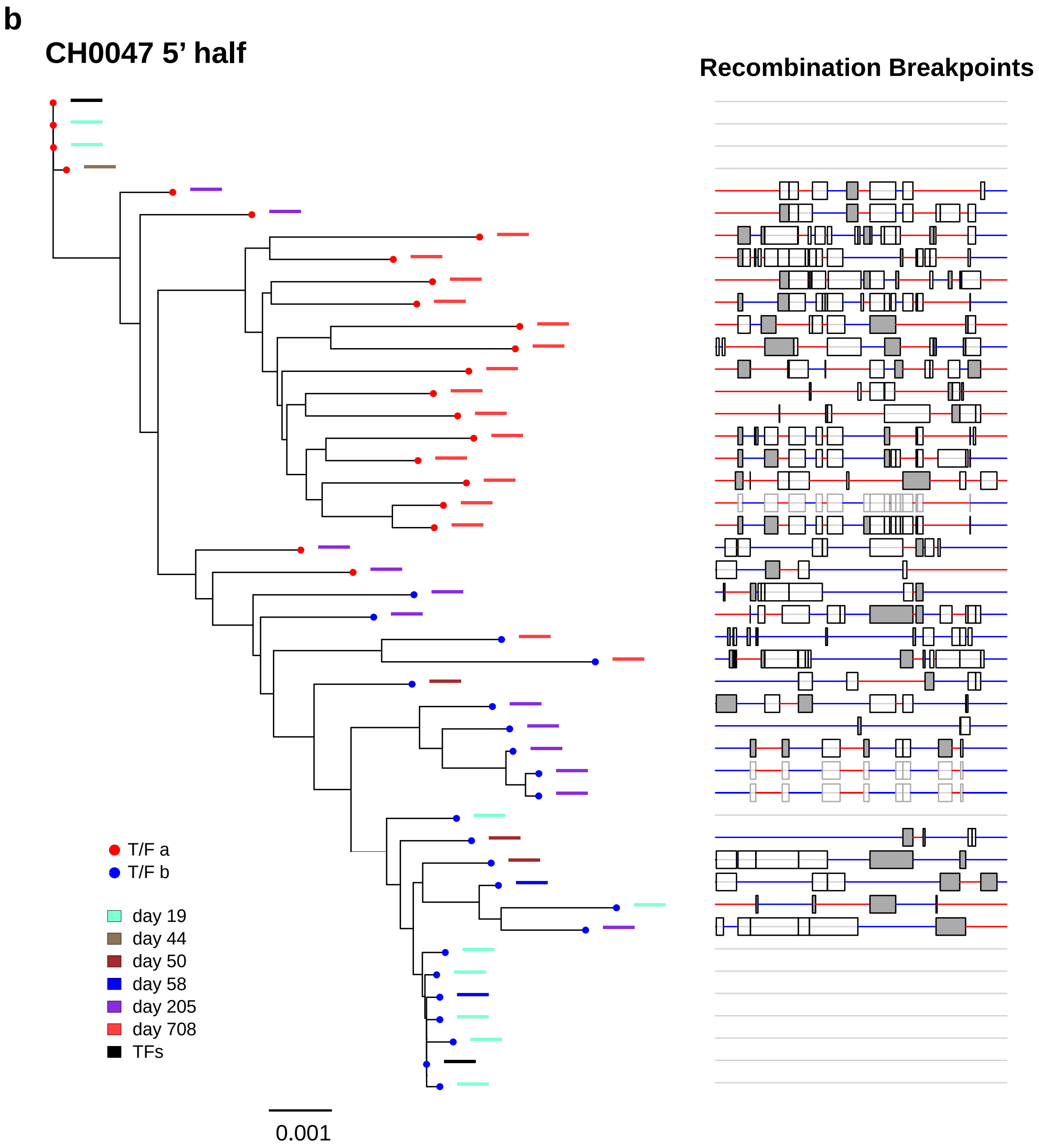


**Supplementary Figure 6**. **RAPR output figures for CH0047 3’ half genome (a) and 5’ half genome (b).**

**a**

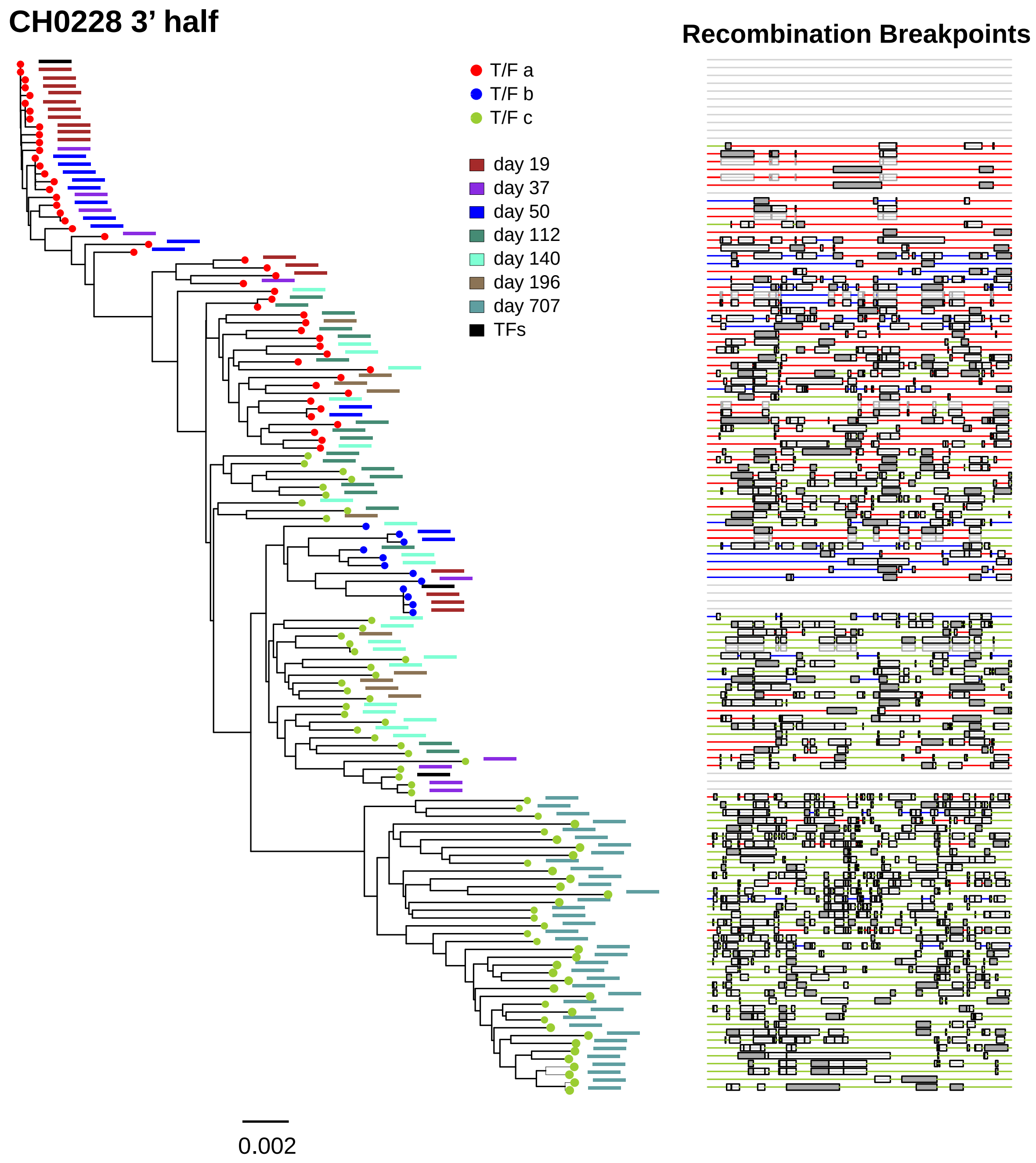

CH0228 3' half
T/F a
T/F b
T/F c
day 19
day 37
day 50
day 112
day 140
day 196
day 707
TFs
0.002
Recombination Breakpoints

**b**

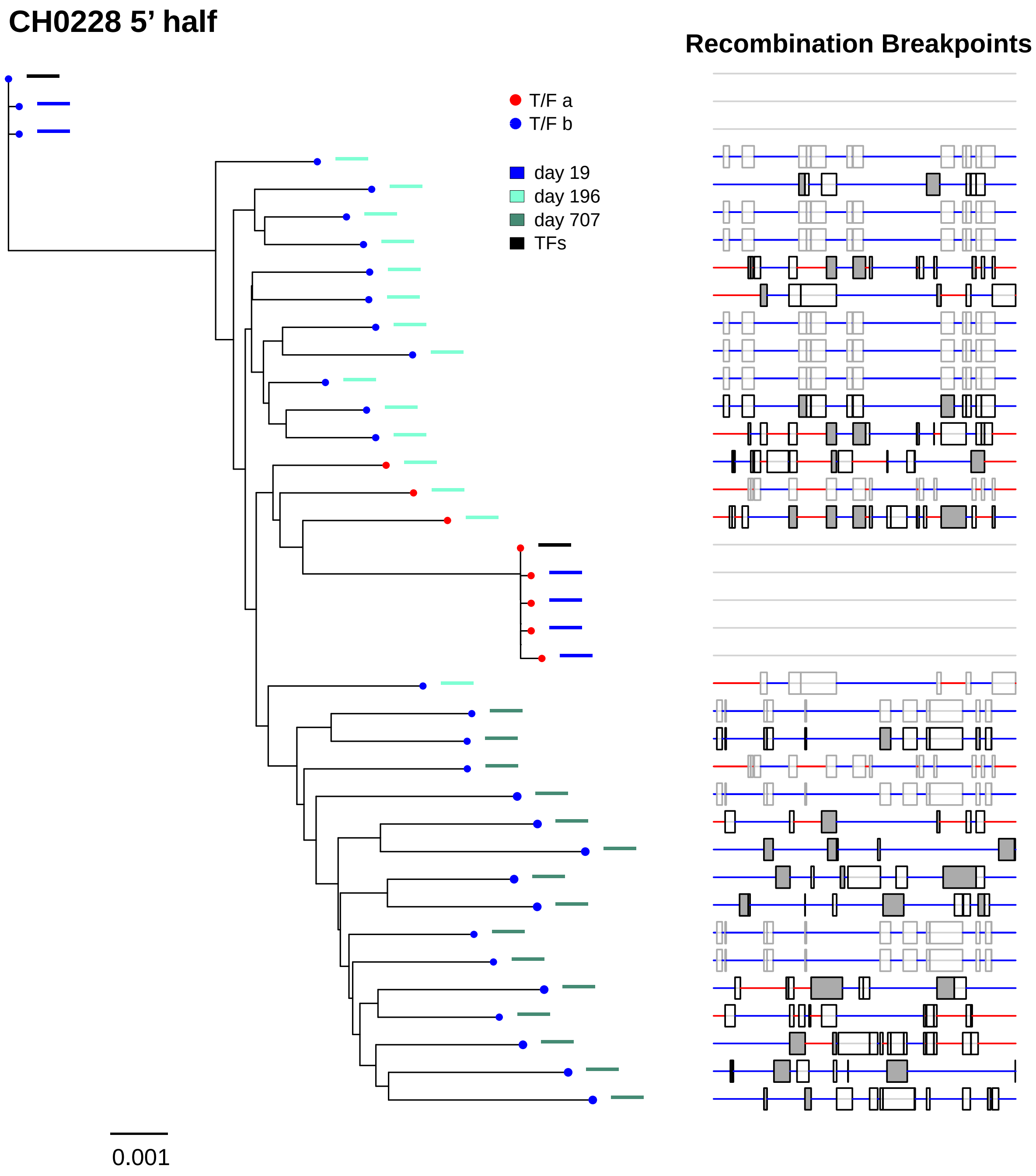


**Supplementary Figure 7. RAPR output figures for CH0228 3' half genome (a) and 5' half genome (b).**

**a**

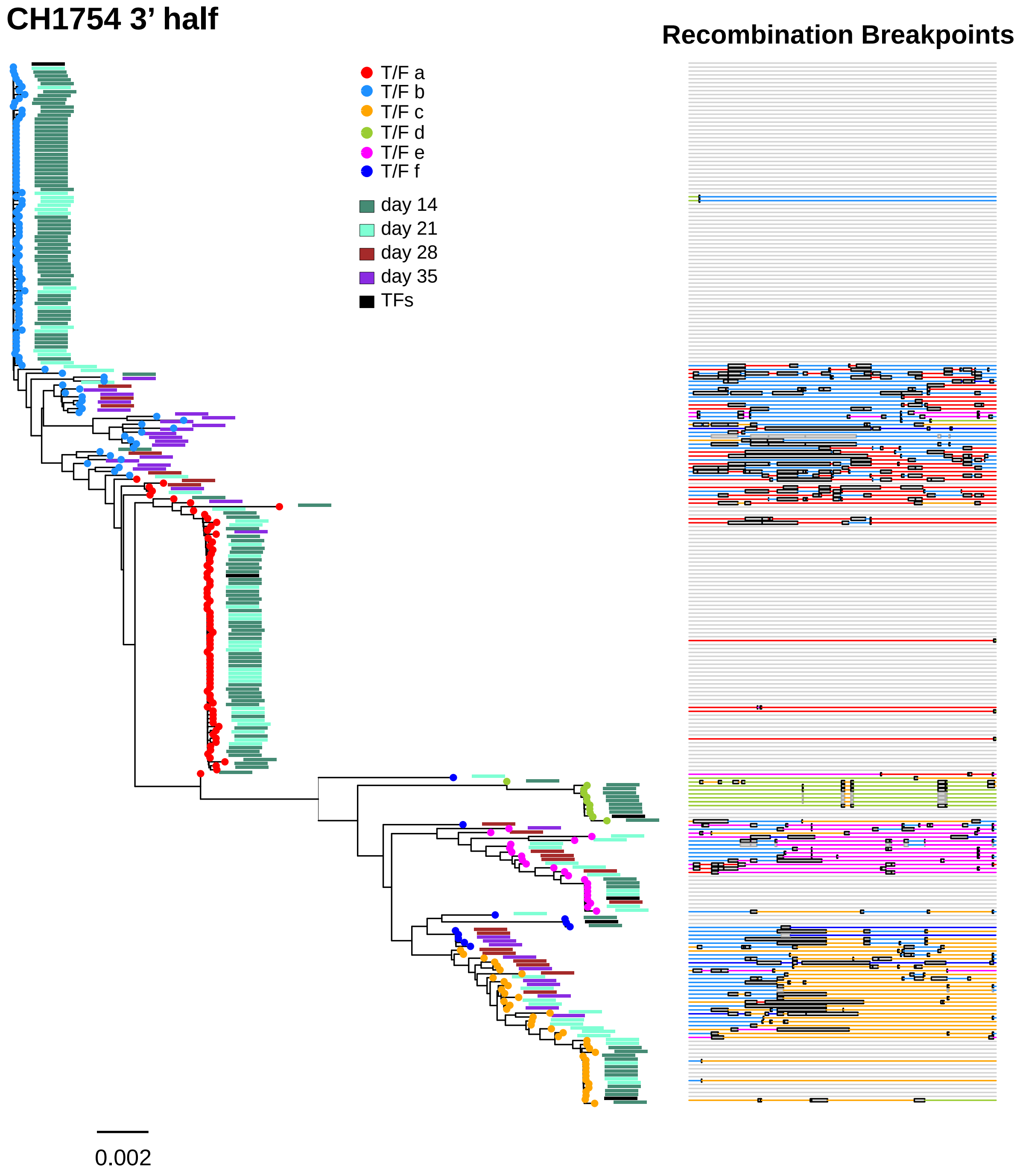
CH1754 3' half
Recombination Breakpoints
T/F a
T/F b
T/F c
T/F d
T/F e
T/F f
day 14
day 21
day 28
day 35
TFs
0.002

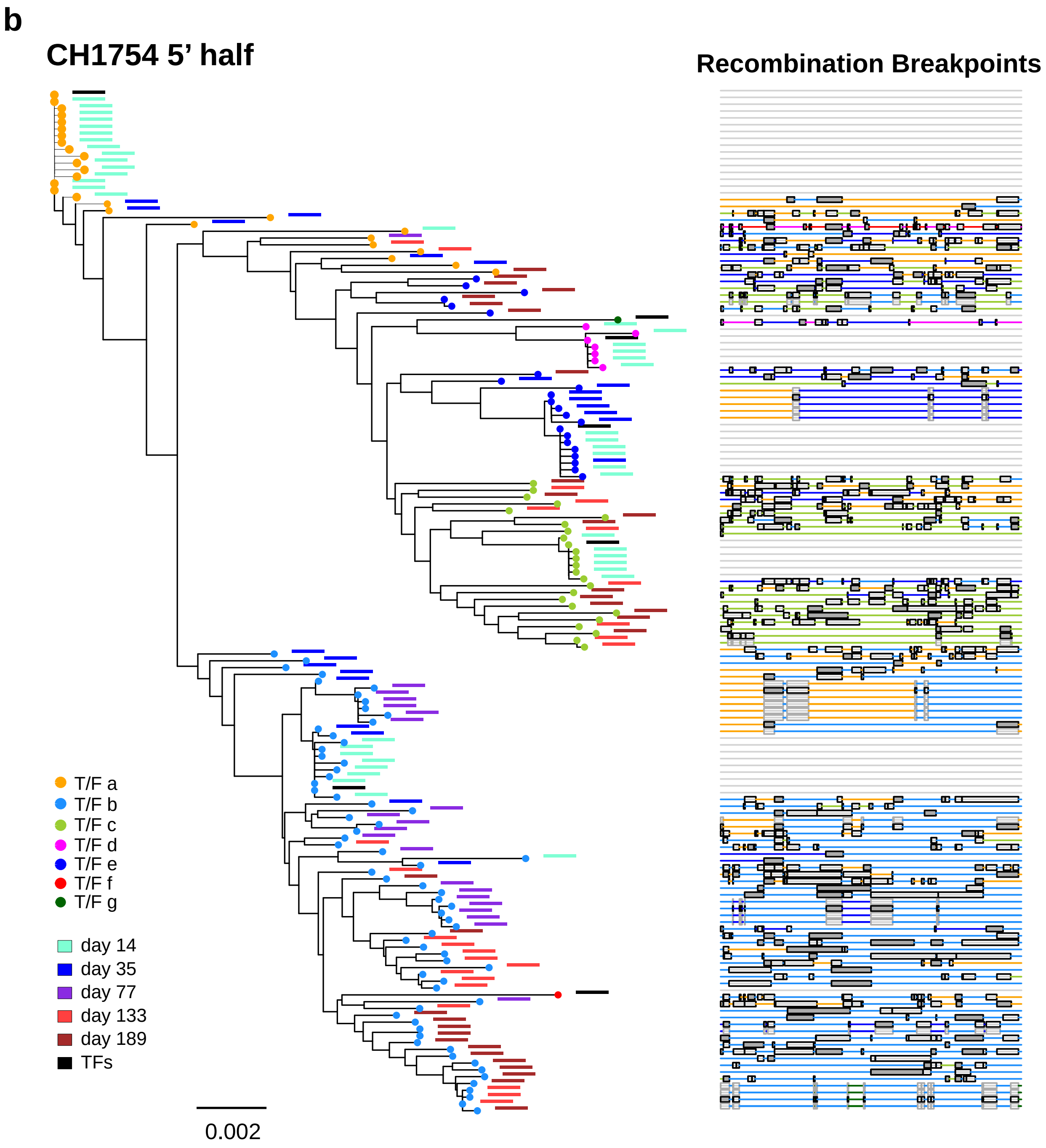


**Supplementary Figure 8. RAPR output figures for CH1754 3' half genome (a) and 5' half genome (b).**

**a**

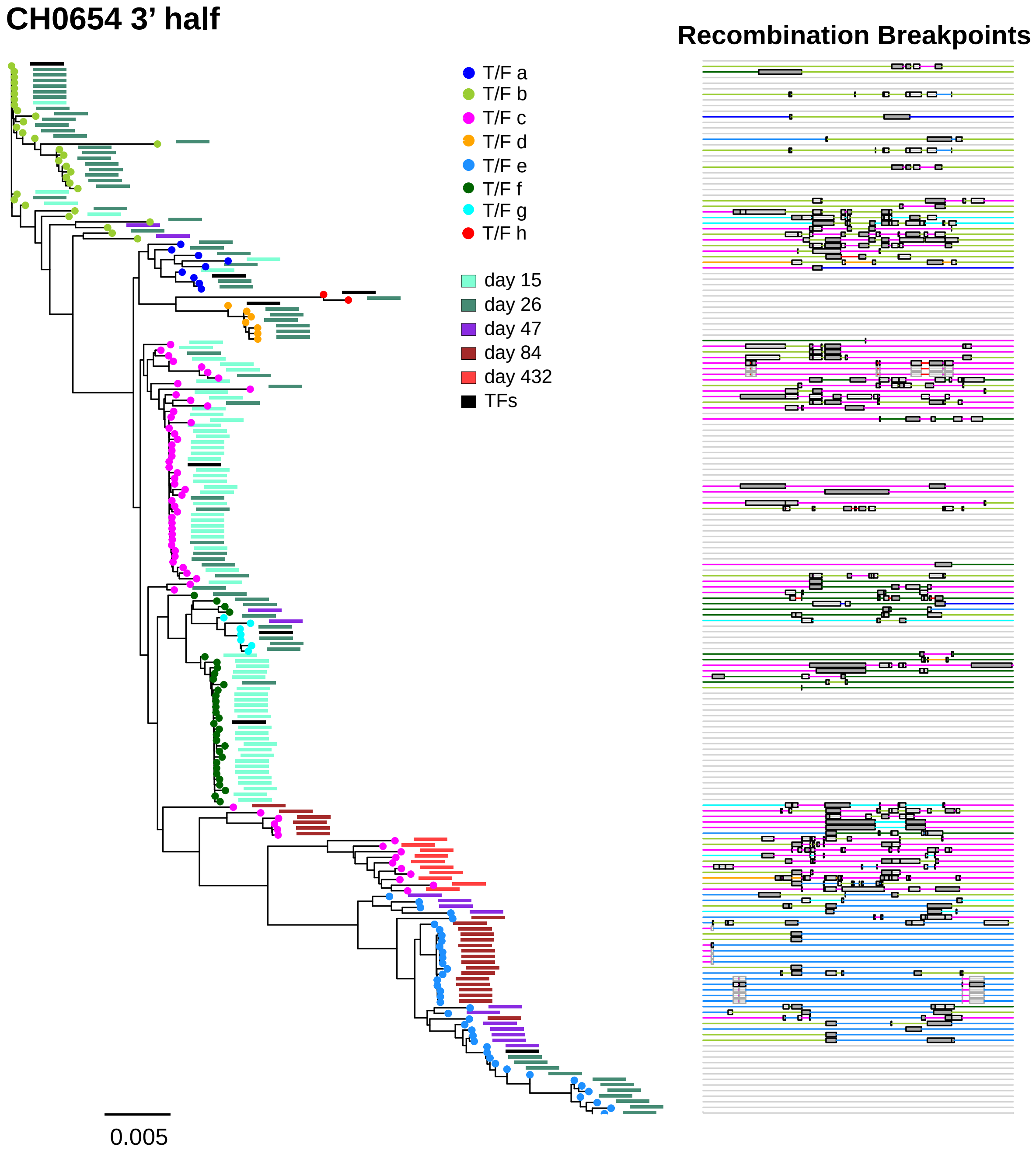
CH0654 3' half
Recombination Breakpoints
T/F a
T/F b
T/F c
T/F d
T/F e
T/F f
T/F g
T/F h
day 15
day 26
day 47
day 84
day 432
TFs
0.005

**b**

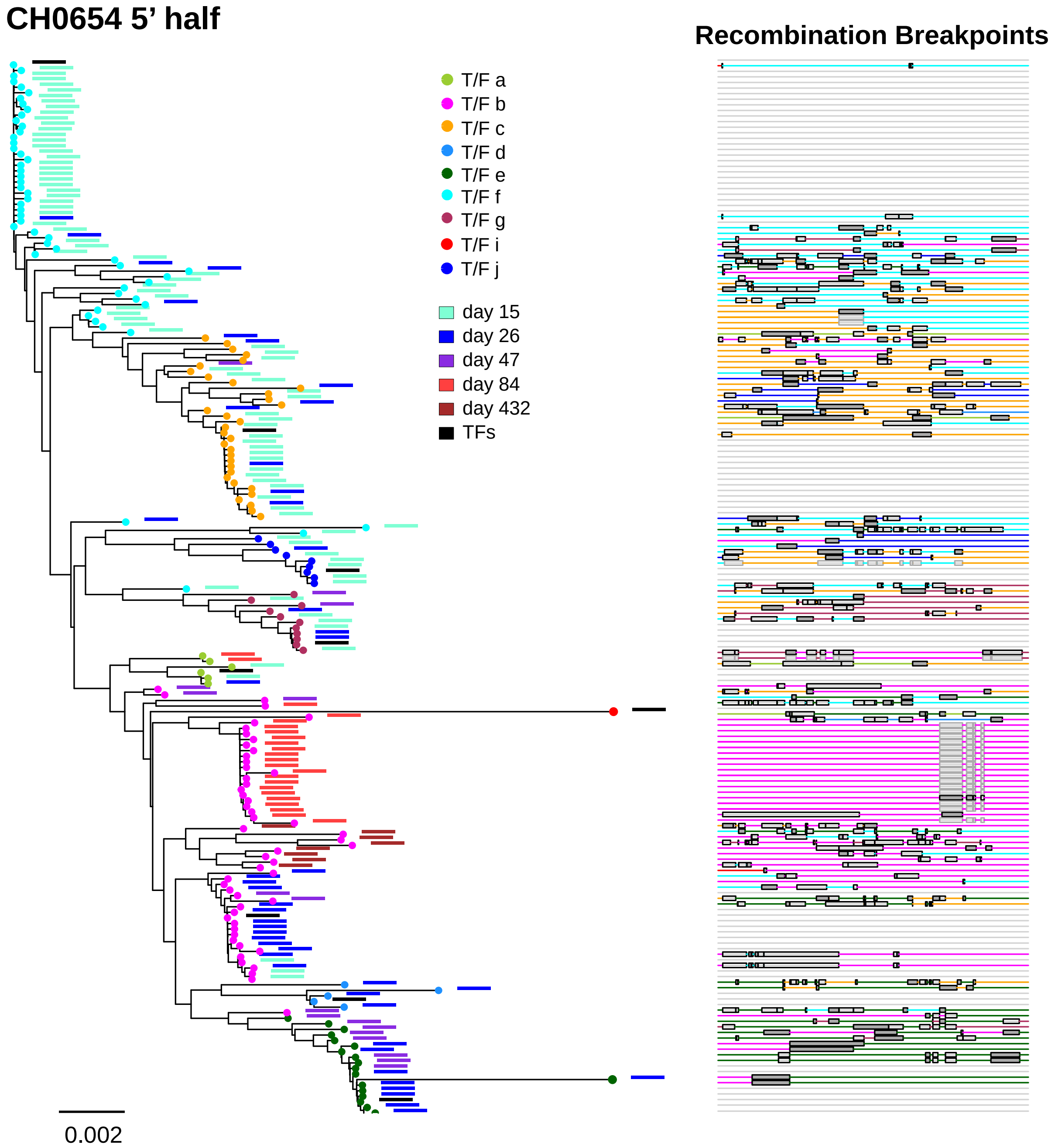


**Supplementary Figure 9. RAPR output figures for CH0654 3' half genome (a) and 5' half genome (b).** The last time point of CH0654 (day 432) was excluded from all analysis due to the initiation of ART from day 112 after infection.

# CH1244 3’ half

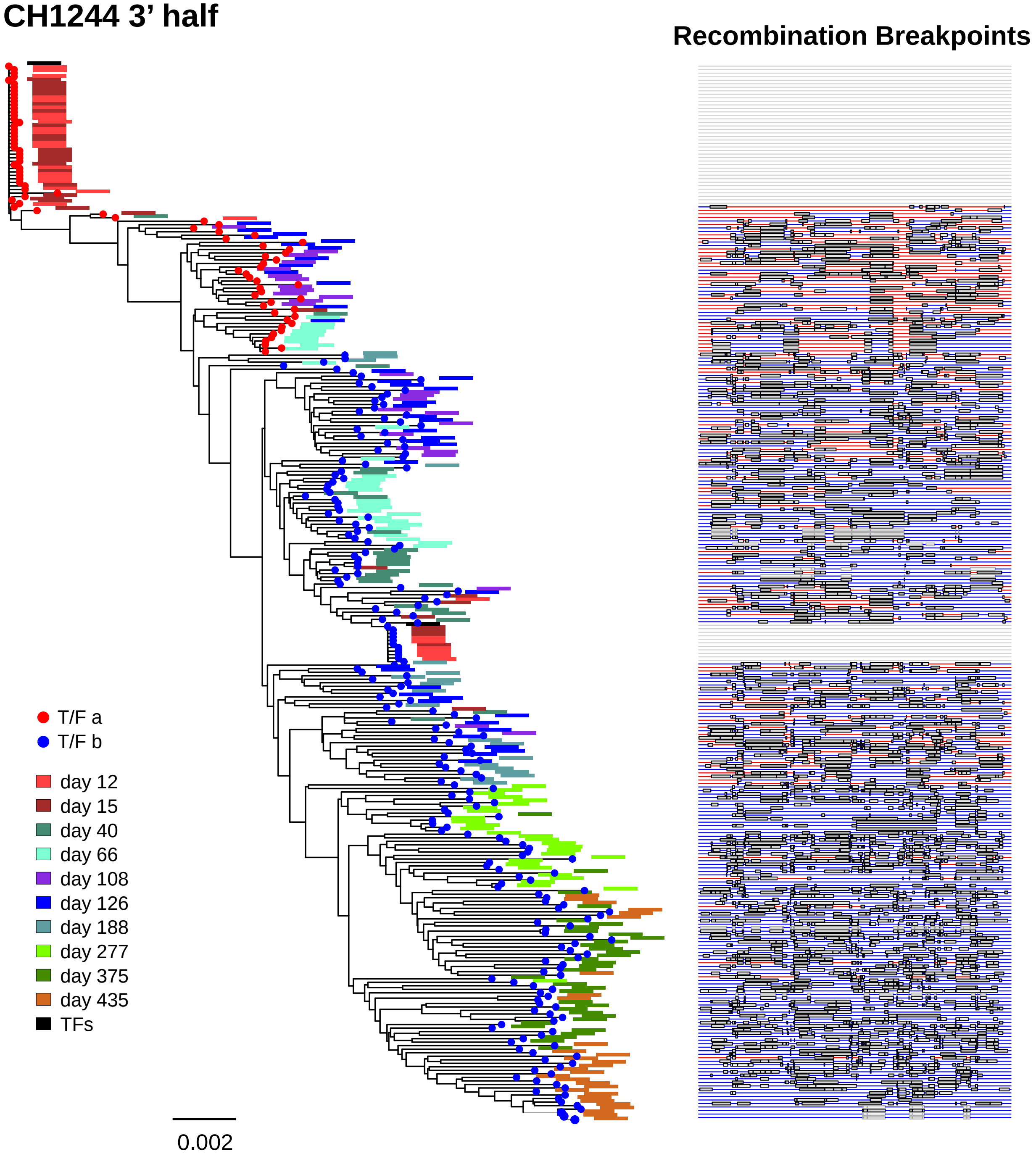


**Supplementary Figure 10. RAPR output figure for CH1244 3’ half genome.**

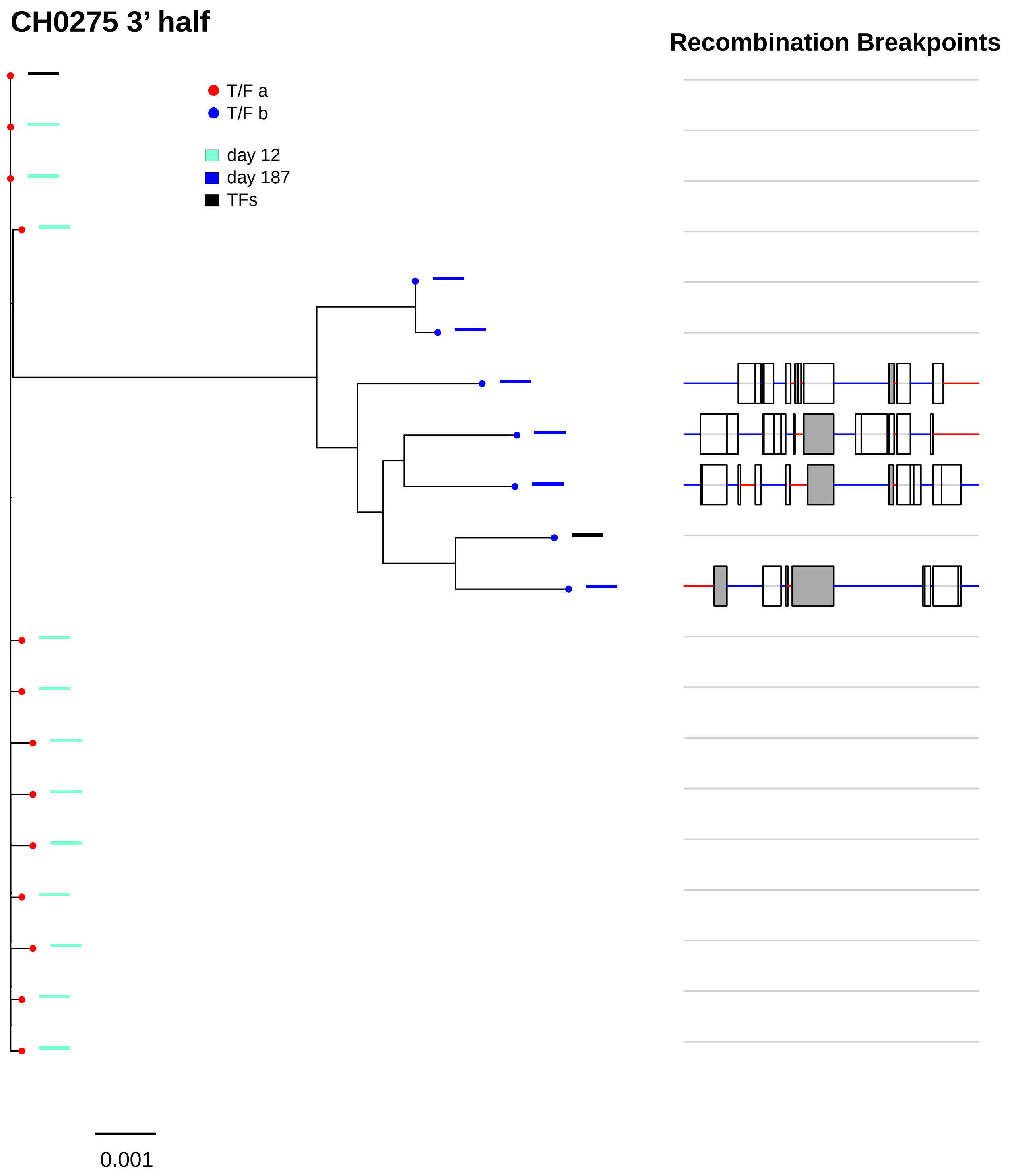


**Supplementary Figure 11**. **RAPR output figure for CH0275 3’ half genome.**

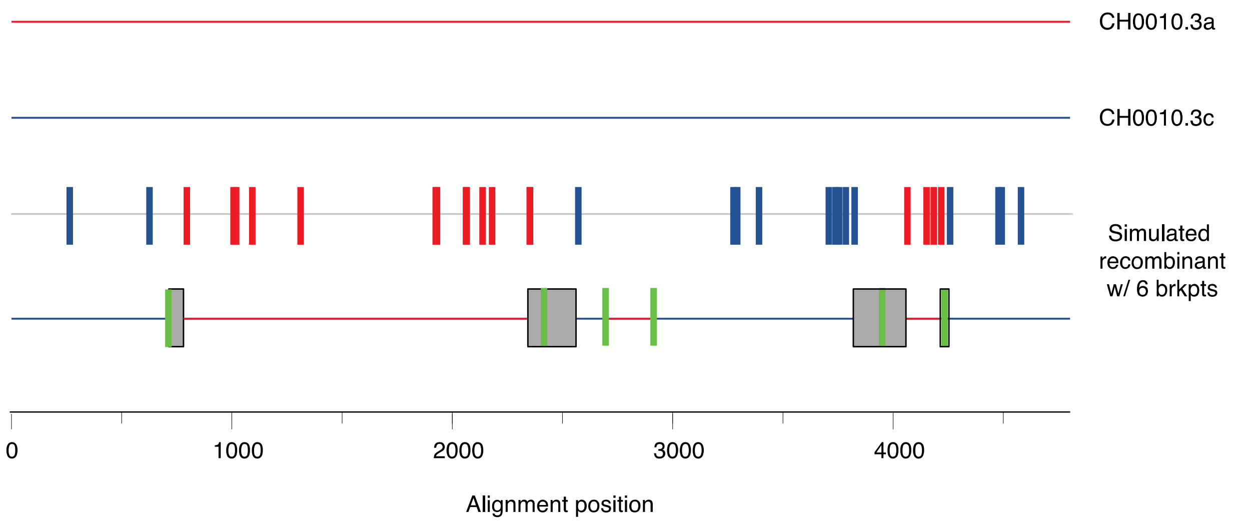


**Supplementary Figure 12**. **Example of true and RAPR detected breakpoints on a simulated recombinant.** We created an artificial recombinant from subject CH0010 T/Fs a and c (shown as horizontal lines in red and blue, respectively) and tested it through RAPR. The third and fourth lines represent two views of the recombinants: the third line shows the mutations the recombinant inherited from either parent (red when they match T/F a and blue when they match T/F c). The fourth line shows the true breakpoints in green and the boxes show the intervals where RAPR detects a breakpoint. As one can see, RAPR correctly detects 4 out of 6 breakpoints. The two undetected ones fall in a region where the two parental strains are identical and therefore the program could not distinguish the switch from blue to red to blue again. Therefore, breakpoints happening in regions of parental homology are likely to go undetected, especially when they happen in pairs as illustrated in this example.

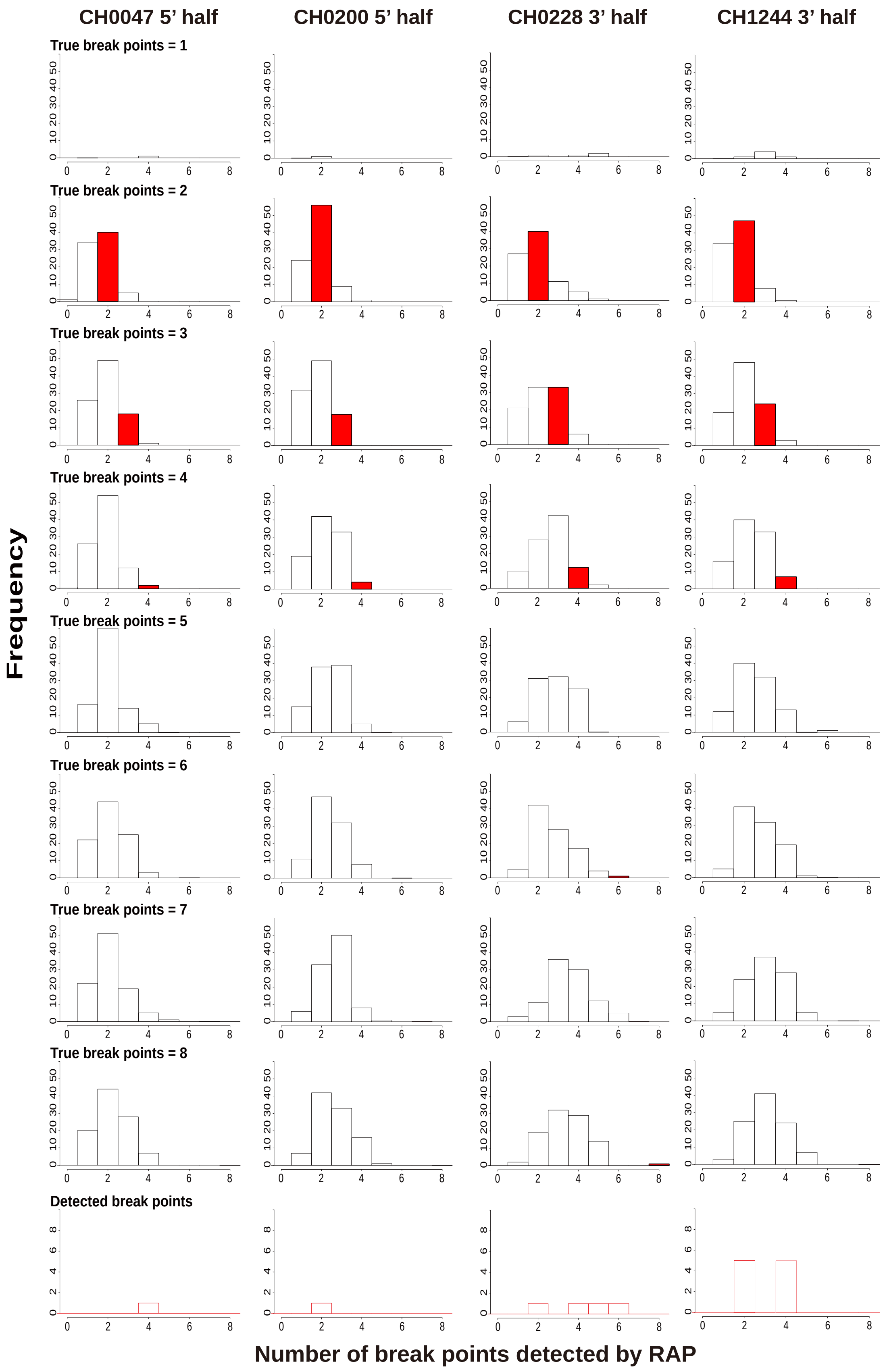

CH0047 5' half
CH0200 5' half
CH0228 3' half
CH1244 3' half
True break points = 1
True break points = 2
True break points = 3
True break points = 4
True break points = 5
True break points = 6
True break points = 7
True break points = 8
Detected break points
Frequency
Number of break points detected by RAP

**Supplementary Figure 13**. **Number of statistically significant breakpoints detected by RAPR on artificially generated recombinants.** Sequences of the 5' half genome of subjects CH0047 and CH0200, and the 3' half genome of subjects CH0228 and CH1244 from the first time point sequences were used for analysis. Out of all alignments in this study, these four were the only ones not to present any recombinant of recombinant at the first time point sampled. For each of the four alignments, we generated 100 artificial recombinants for each breakpoint between 1 and 8 and then ran RAPR on the resulting sequences. Here we show the frequency counts of the significant breakpoints detected by RAPR when the true breakpoints were 1 (top four panels) all the way to 8 (second to last row of panels). The last row of panels show the frequency of the breakpoints detected in the actual data (first time point only). Bars representing the number of times the true break points were detected are highlighted in red. See Materials and Methods for description on how statistical significance was determined and how the artificial recombinants were generated.

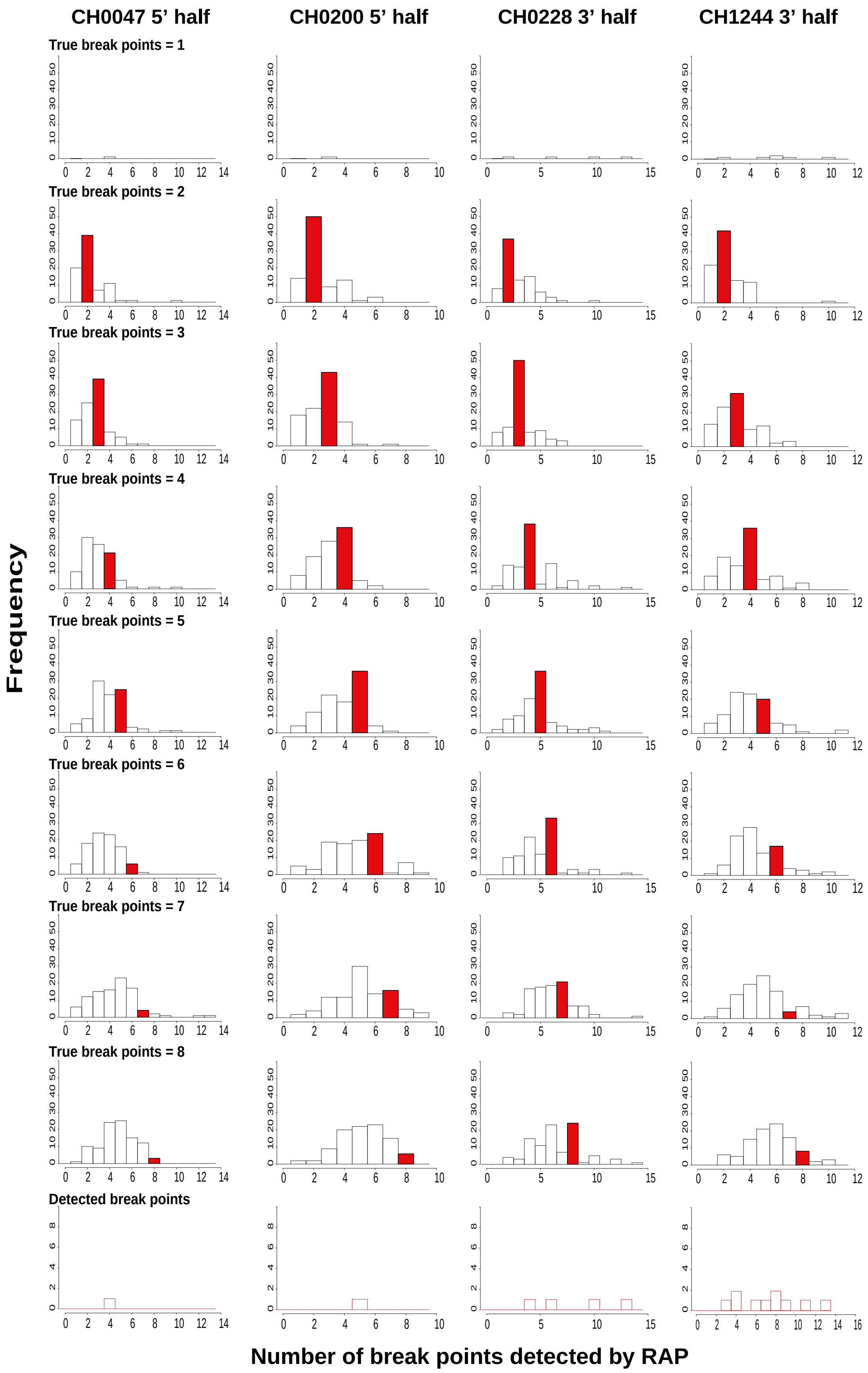
CH0047 5’ half
CH0200 5’ half
CH0228 3’ half
CH1244 3’ half
True break points = 1
True break points = 2
True break points = 3
True break points = 4
True break points = 5
True break points = 6
True break points = 7
True break points = 8
Detected break points
Frequency
Number of break points detected by RAP

**Supplementary Figure 14**. **Total number of breakpoints detected by RAPR on artificially generated recombinants from first time point sequences from the 5' half genome of subjects CH0047 and CH0200, and the 3' half genome of subjects CH0228 and CH1244.** Out of all alignments in this study, these four were the only ones not to present any recombinant of recombinant at the first time point sampled. For each of the four alignments, we generated 100 artificial recombinants for each breakpoint between 1 and 8 and then ran RAPR on the resulting sequences. Here we show the frequency counts of the significant breakpoints detected by RAPR when the true breakpoints were 1 (top four panels) all the way to 8 (second to last row of panels). Bars representing the number of times the true break points were detected are highlighted in red. The last row of panels show the frequency of the breakpoints detected in the actual data (first time point only).

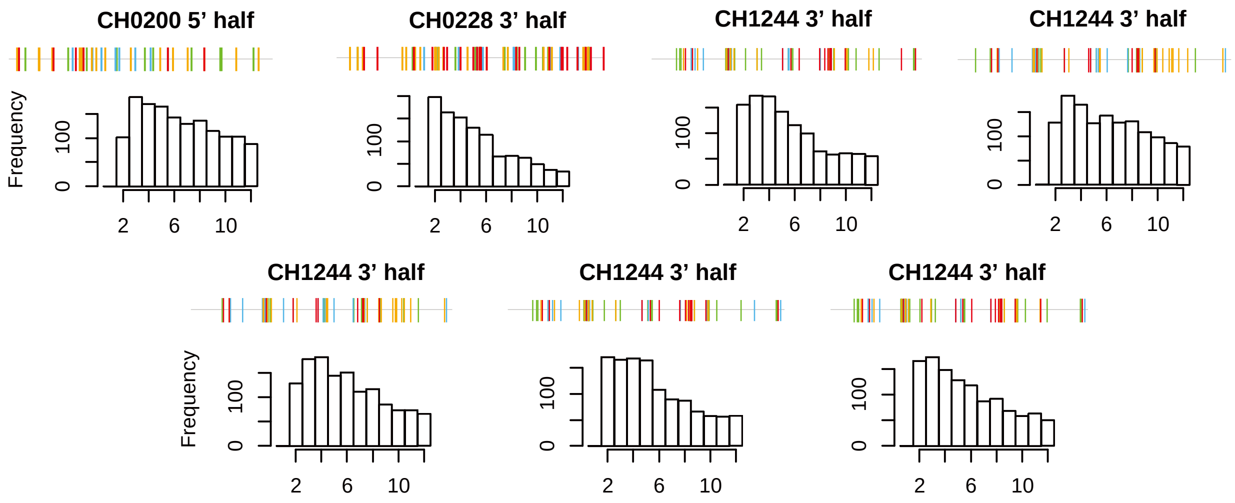


True Break Points, RAP detects 2

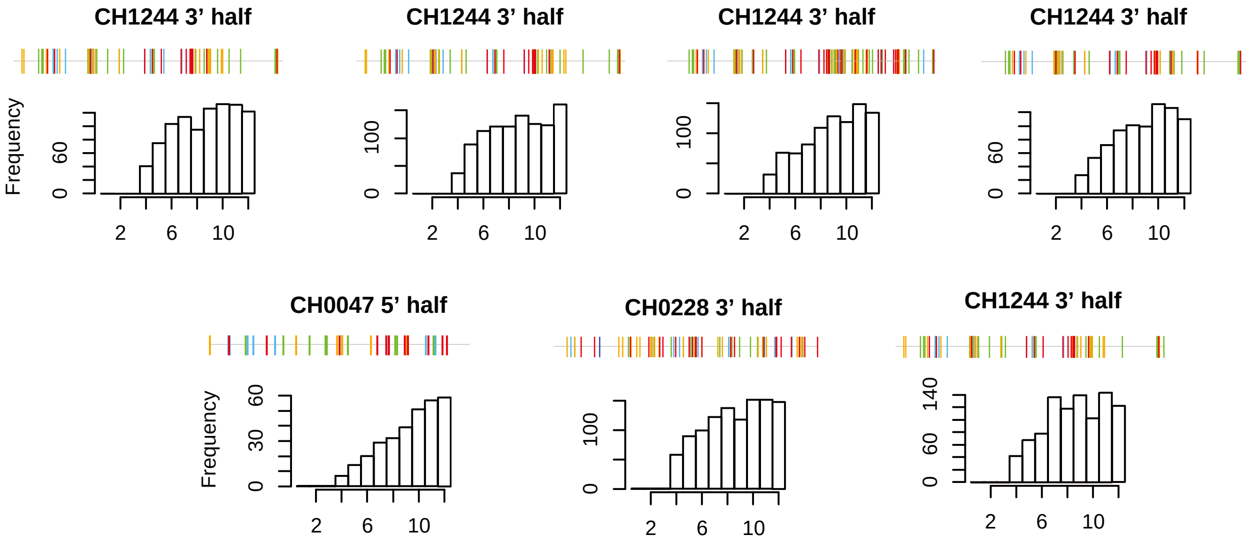


True Break Points, RAP detects 4

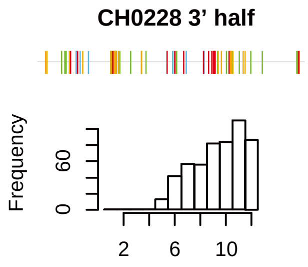


True Brk Points, RAP detects 5

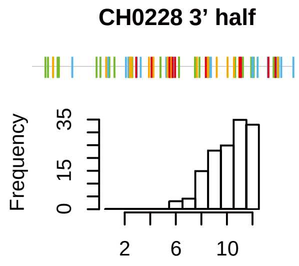


True Brk Points, RAP detects 6

**Supplementary Figure 15**. **Each panel shows the frequency counts of true break points from artificial recombinants recreated from parental strains that in each subject were detected by RAPR to have generated an early time point recombinant.** We identified four subjects where no recombinant of recombinants was detected at the early time points. For each recombinant in each of the four subjects, we generated 5,000 artificial recombinants from the same parental strains and then plotted the frequency counts of the true break points of the artificial recombinants for which RAPR detected the same number of breakpoints as it found in the observed recombinant.

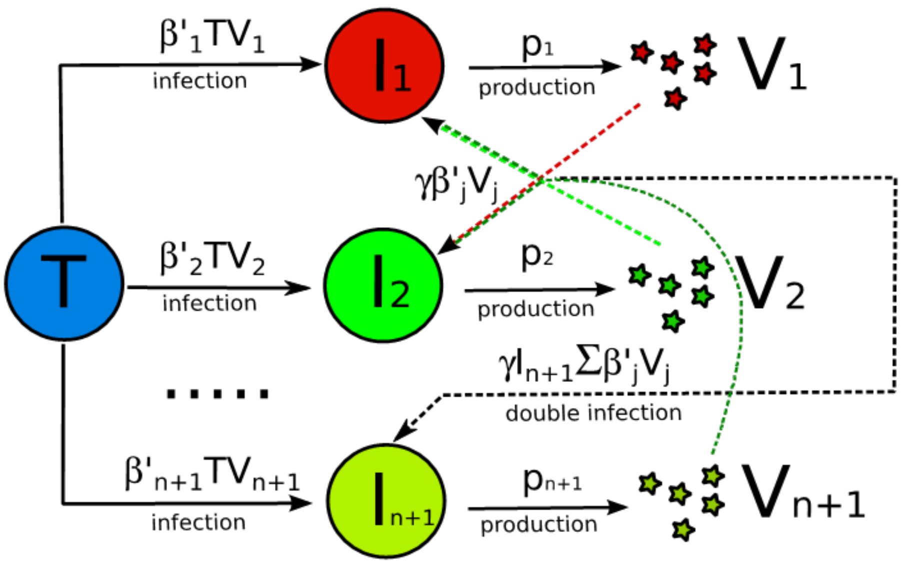


**Supplementary Figure 16**. **Conceptual diagram of the HIV dynamics and evolutionary model used to determine the replacement half-time by recombinants.** In our mathematical model, uninfected target cells $T$ become infected with T/F viruses or recombinant strains at a rate $\beta_j'$ where $j$=1…$n$+1 (where $n$ is the number of T/Fs, and $j$=$n$+1 represents the recombinant sequences). Cells infected with one viral strain can be coinfected with other viral strains, and such coinfection leads to the generation of the recombinant virus. Coinfection occurs at a reduced rate $\gamma\beta_j'$. We expect $\gamma<1$ due to down-regulation of receptors CD4 and/or CCR5 on infected cells. The rate of virus production by infected cells is denoted as $p_i$. Viruses and infected cells are cleared at the rate $c$ and $\delta_j$, respectively (not depicted in the cartoon), $I_j$ is the density of cells infected with T/F viruses ($j$=1…$n$), $I_{n+1}$ is the density of cells infected with the recombinant virus, $V_j$ is the density of viral particles. All recombinant viruses are assumed to have the same replication and death-related parameters, but these may be different from parameters of the T/F viruses.

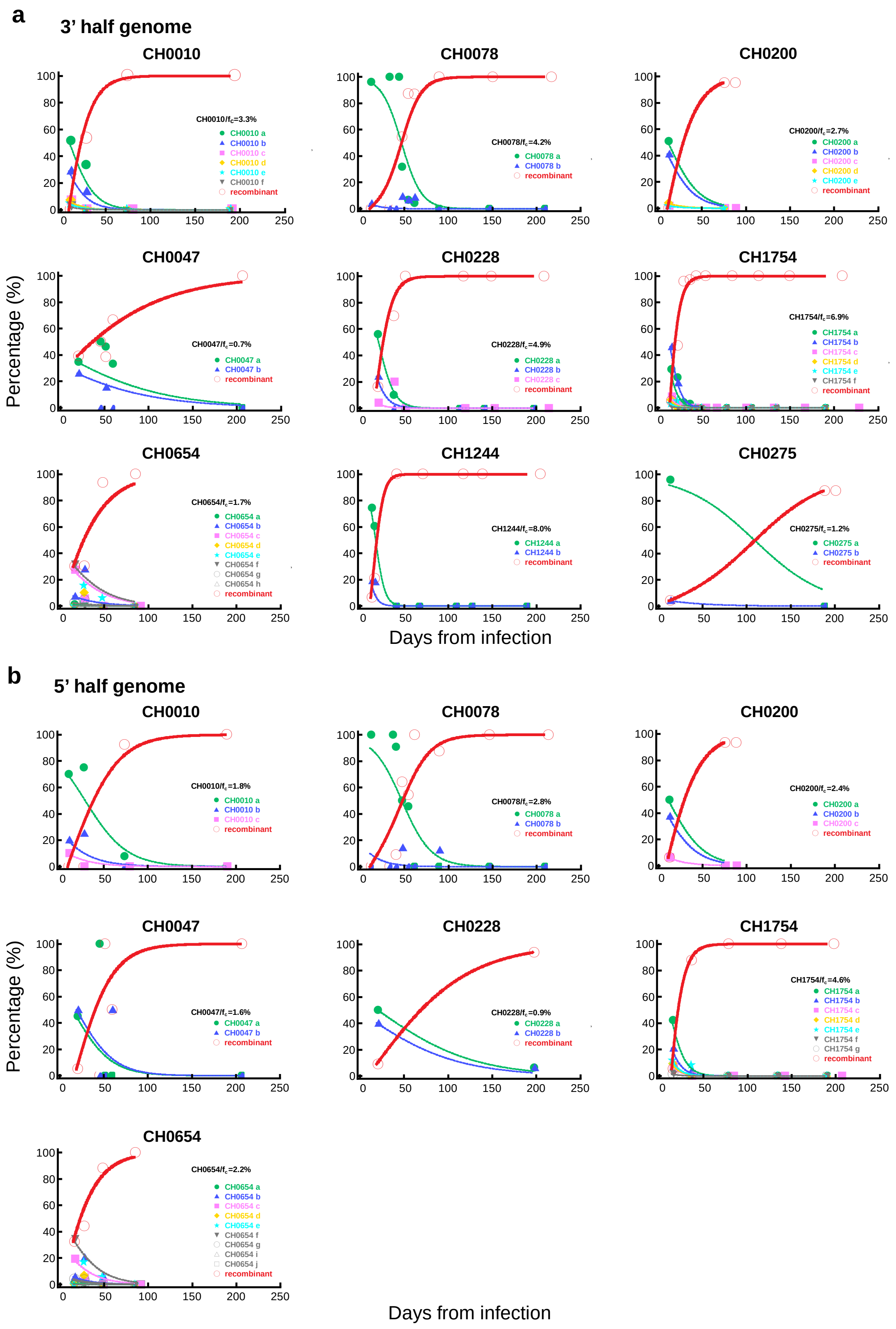

a
3' half genome
CH0010
CH0010/fc=3.3%
CH0010 a
CH0010 b
CH0010 c
CH0010 d
CH0010 e
CH0010 f
recombinant
CH0078
CH0078/fc=4.2%
CH0078 a
CH0078 b
recombinant
CH0200
CH0200/fc=2.7%
CH0200 a
CH0200 b
CH0200 c
CH0200 d
CH0200 e
recombinant
CH0047
CH0047/fc=0.7%
CH0047 a
CH0047 b
recombinant
CH0228
CH0228/fc=4.9%
CH0228 a
CH0228 b
CH0228 c
recombinant
CH1754
CH1754/fc=6.9%
CH1754 a
CH1754 b
CH1754 c
CH1754 d
CH1754 e
CH1754 f
recombinant
CH0654
CH0654/fc=1.7%
CH0654 a
CH0654 b
CH0654 c
CH0654 d
CH0654 e
CH0654 f
CH0654 g
CH0654 h
recombinant
CH1244
CH1244/fc=8.0%
CH1244 a
CH1244 b
recombinant
CH0275
CH0275/fc=1.2%
CH0275 a
CH0275 b
recombinant
Percentage (%)
Days from infection
b
5' half genome
CH0010
CH0010/fc=1.8%
CH0010 a
CH0010 b
CH0010 c
recombinant
CH0078
CH0078/fc=2.8%
CH0078 a
CH0078 b
recombinant
CH0200
CH0200/fc=2.4%
CH0200 a
CH0200 b
CH0200 c
recombinant
CH0047
CH0047/fc=1.6%
CH0047 a
CH0047 b
recombinant
CH0228
CH0228/fc=0.9%
CH0228 a
CH0228 b
recombinant
CH1754
CH1754/fc=4.6%
CH1754 a
CH1754 b
CH1754 c
CH1754 d
CH1754 e
CH1754 f
CH1754 g
recombinant
CH0654
CH0654/fc=2.2%
CH0654 a
CH0654 b
CH0654 c
CH0654 d
CH0654 e
CH0654 f
CH0654 g
CH0654 i
CH0654 j
recombinant
Percentage (%)
Days from infection

**Supplementary Figure 17. Rapid dominance of recombinants in the viral population in each individual can be explained by a simple mathematical model assuming infrequent coinfection of target cells by two viruses.** The solution to the basic mathematical model (see Methods) was fitted to recombinant frequency for each subject using maximum likelihood. Each graph shows the plot of the dynamics of the accumulation of recombinants (red circles are data and red lines are model predictions) and the loss of T/F viruses (markers are data and thin dashed lines are model predictions) in each individual in both the 3' half genome **(a)** and 5' half genome **(b)**. The parameter $f_c$ denotes the predicted frequency of coinfection of target cells by two different viruses. Note that the loss of the T/F viruses was not directly fitted and is predicted from the model given the rate of coinfection $\gamma\beta I$.

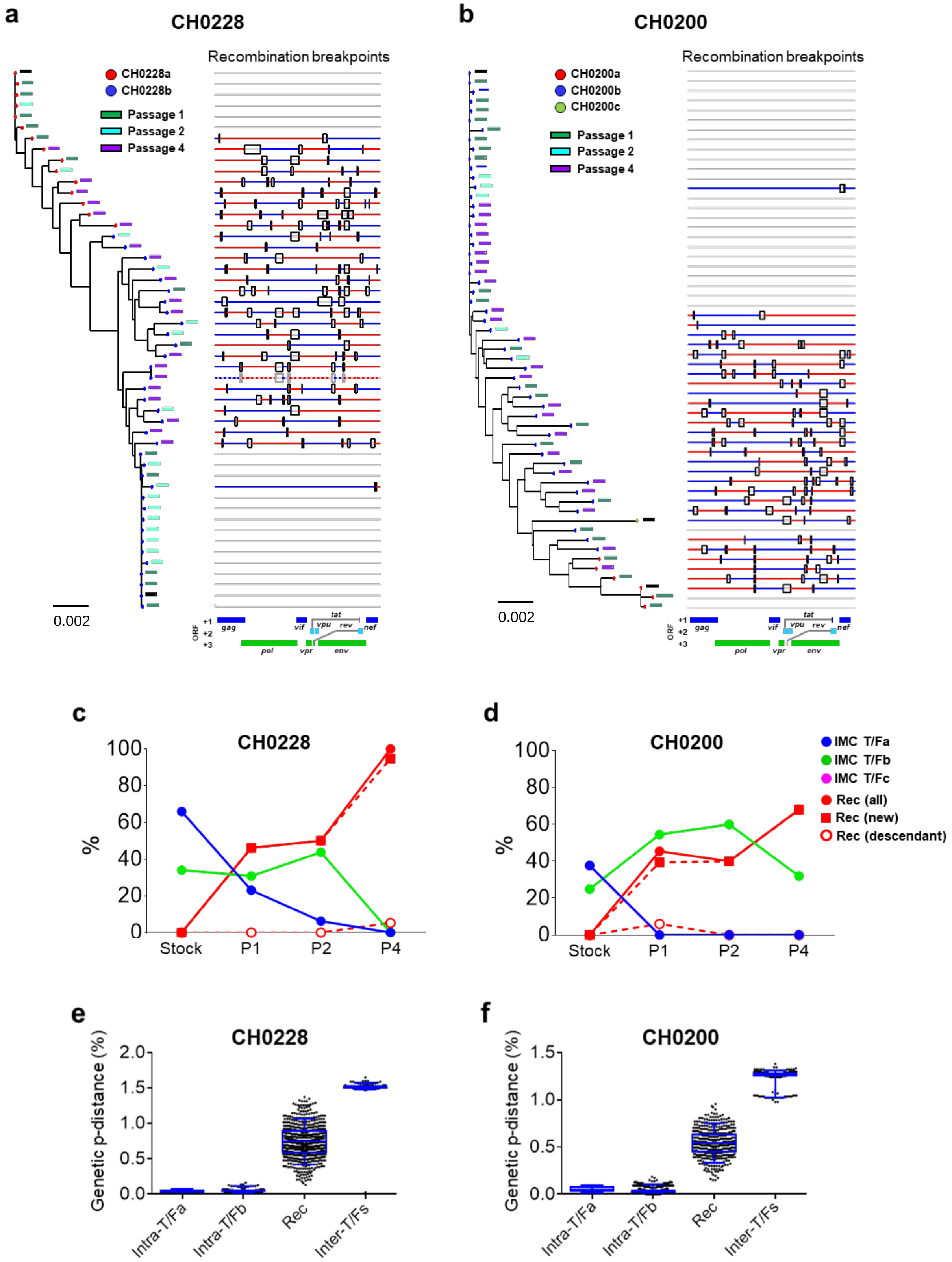
a
CH0228
Recombination breakpoints
CH0228a
CH0228b
Passage 1
Passage 2
Passage 4
0.002
b
CH0200
Recombination breakpoints
CH0200a
CH0200b
CH0200c
Passage 1
Passage 2
Passage 4
0.002
ORF
gag
vif
vpu
tat
rev
nef
pol
vpr
env
c
CH0228
%
Stock
P1
P2
P4
d
CH0200
IMC T/Fa
IMC T/Fb
IMC T/Fc
Rec (all)
Rec (new)
Rec (descendant)
e
CH0228
Genetic p-distance (%)
Intra-T/Fa
Intra-T/Fb
Rec
Inter-T/Fs
f
CH0200

**Supplementary Figure 18. Evolution of viral recombination in infected CD4+ T cells *in vitro.*** Frequencies of the parental viruses and their recombinants were determined in the inoculum stocks and at passages 1, 2, and 4 for CH0228 (**a** and **b**) and CH0200 (**c** and **d**). The frequencies of each variant in the inoculum were determined by PASS. NFLG genomes at each passage were obtained by SGA sequencing. For each sequence (leaf) in the tree, color dots denote the closest T/F (by branch length). Colored bars at each sequence (leaf) instead indicate the time point (passage). Recombination breakpoints were determined by RAPR for CH0200 and CH0228. Each line represents a sequence. Colored intervals in each recombinant sequence are according to the parental T/F as shown in Fig. 1. Recombinants and their descendants were identified for the NFLG sequences of CH0200 **(b)** and CH0228 **(d)** using RAPR. The percentages of T/Fs (colored dots), recombinants (red dot), *de novo* recombinants (red square), and recombinant descendants (red circle) were determined in the virus population at each time passage. Pairwise genetic distances (p-distance) within lineages, inter-lineages and within recombinants were calculated for CH0228 **(e)** and CH0200 **(f)** by combining all viral sequences from all passages.

Resistance-conferring positions in recombination events
Subject CH0505

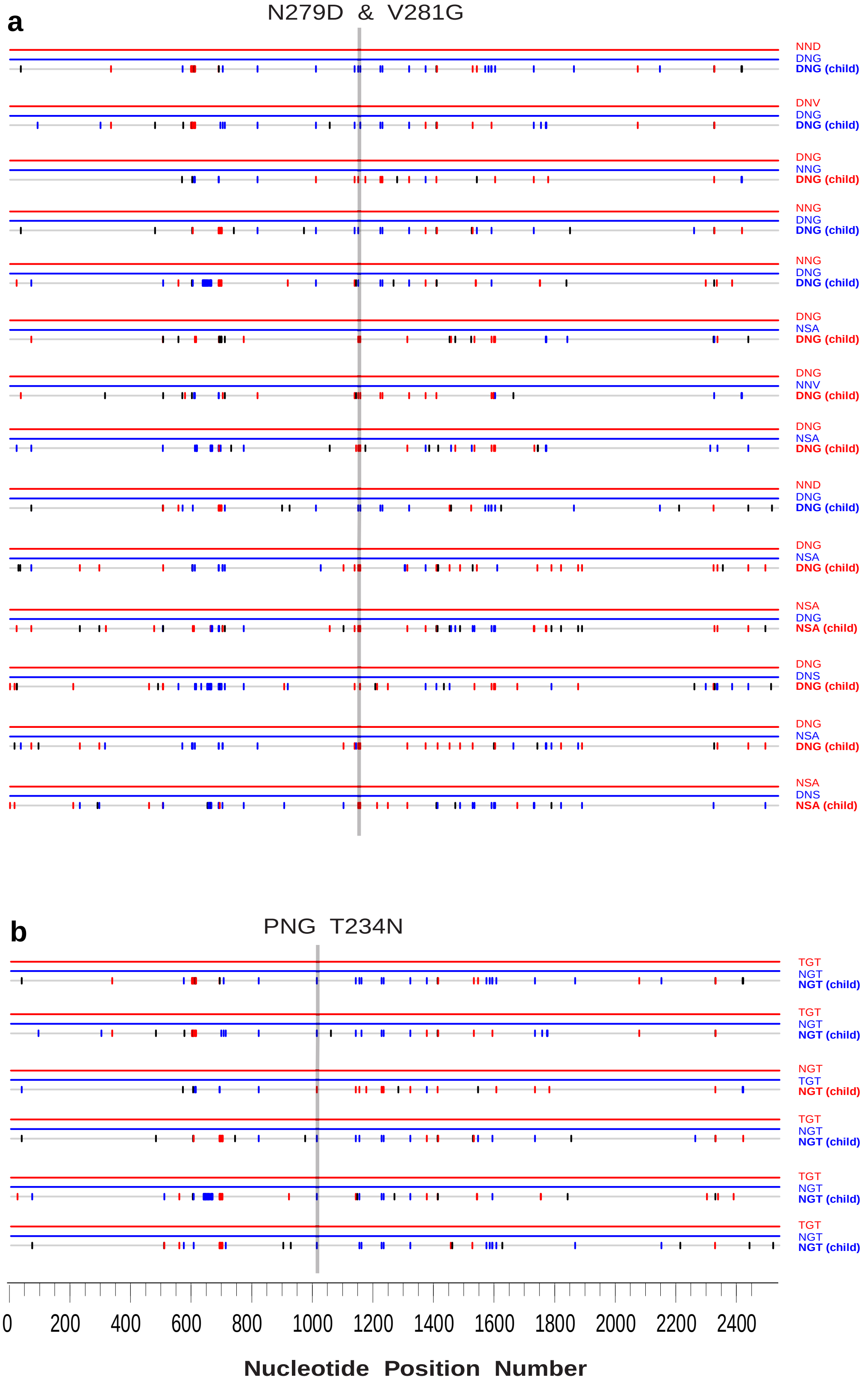

**Supplementary Figure 19**. **Parent-recombinant triplets from subject CH0505 showing how resistance-conferring mutations tend to be carried on to the recombinant when parents are heterogeneous. (a)** In the D Loop, we identified two mutations, N279D and V281G that conferred autologous resistance. At position 279, 10 out of 12 times the D substitution was preferred, and at position 281, 9 out of 10 times the G substitution was preferred. Parental strains are shown in red and blue respectively and mutations in the child are either red or blue depending on whether they match the first parent or the second, black if they don't match either one. **(b)** We identified 6 recombination events where the parents were heterogeneous at the glycosylation site 234, and in all 6 instances, the resistance-conferring glycosylation site was carried on to the child.

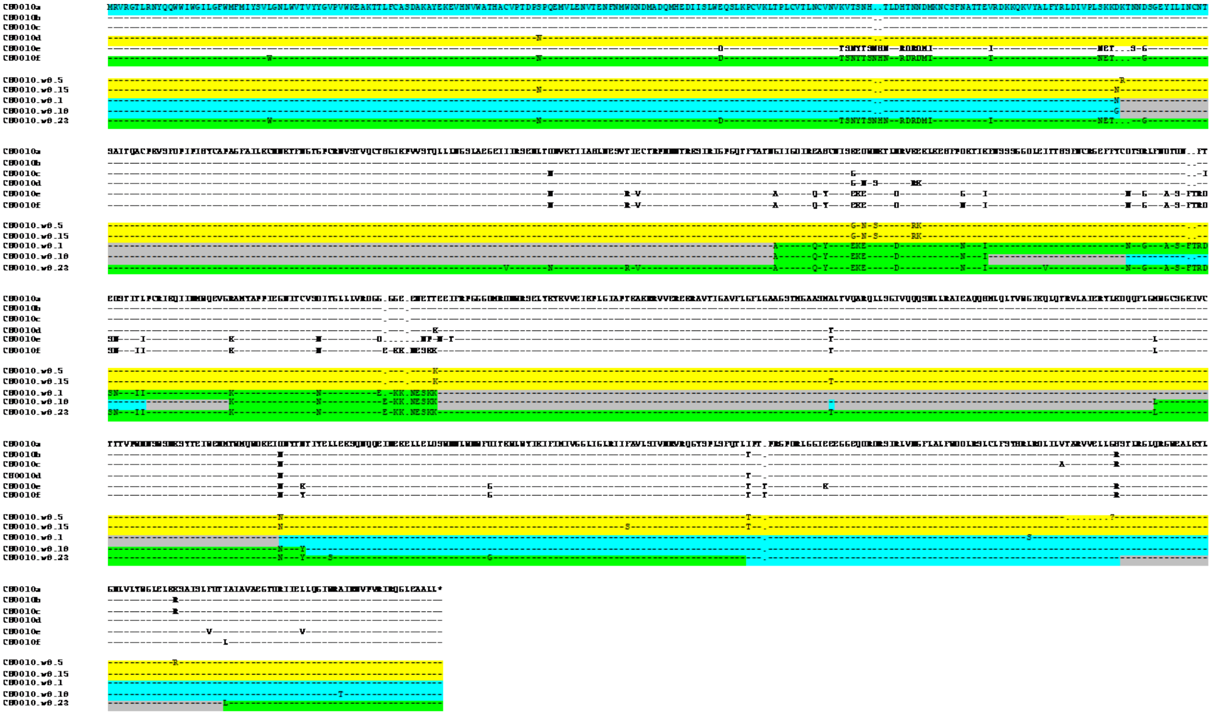

**Supplementary Figure 20. Sequence alignment of T/F viruses and recombinants in CH0010.** Env amino acid sequences from six T/Fs and five week 8 (day 72) variants from subject CH0010 were aligned together. All sequences were compared to CH0010 T/Fa. Sequences originated from T/Fa, T/Fd and T/Ff viruses are shown in blue, yellow and green color, respectively. The possible regions of recombination breakpoints are shown in gray.

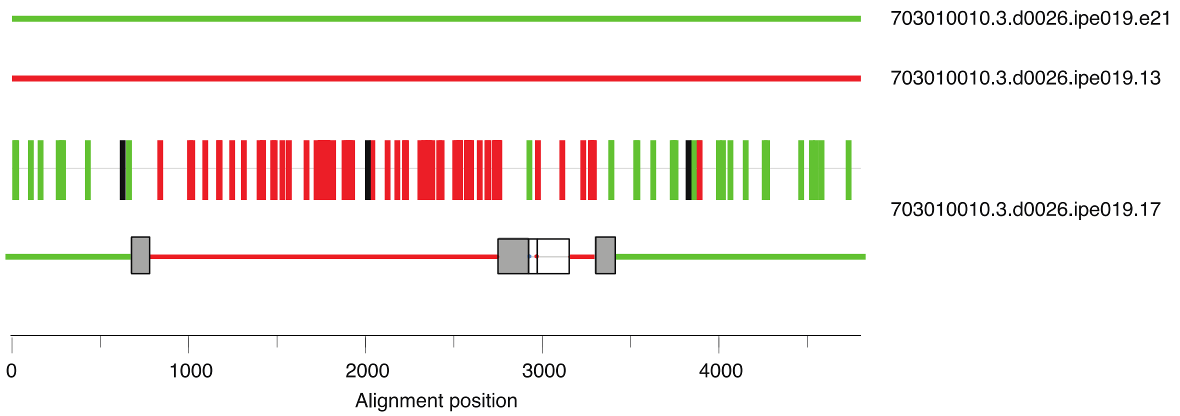


**Supplementary Figure 21. A recombination event from subject CH0010 (3' half genome)**. Sequence 703010010.3.d0026.ipe019.17, originates from parents 703010010.3.d0026.ipe019.e21 and 703010010.3.d0026.ipe019.13, and it's the strongest signal found in the alignment (Runs test p-value 9e-14). Parental strains are represented as straight lines, and the recombinant is represented in two ways: the top representation shows each run from the parental strain as a colored vertical bar (black indicates mutations unique to the recombinant). The bottom representation shows the parental strain as a horizontal segment and the range where the breakpoint is most likely to have happened is shown as a box. Boxes shaded in gray represent statistically significant breakpoints, the others are left empty.

**Supplementary Table 1** The 95% lower CL of the true breakpoints and of the recombination rate per nucleotide given the observed recombinants in the first time points of CH0047.5, CH0200.5, CH0228.3, and CH1244.3.

| Sample | Observed Recombinant | RAP detected number of breakpoints | 95% Lower Confidence Limit | 95% Lower Confidence Limit on rate |
|---|---|---|---|---|
| CH0047.5 | 702010047.5.d0019.ipe037.wg7 | 4 | 6 | $1.3 \times 10^{-3}$ |
| CH0200.5 | 703010200.5.d0011.ipe018.wg10 | 2 | 2 | $1.3 \times 10^{-4}$ |
| CH0228.3 | 703010228.3.d0019.ipe025.e11 | 2 | 2 | $1.3 \times 10^{-4}$ |
| CH0228.3 | 703010228.3.d0019.ipe025.e15 | 4 | 4 | $5.2 \times 10^{-4}$ |
| CH0228.3 | 703010228.3.d0019.ipe025.e29 | 5 | 6 | $9.1 \times 10^{-5}$ |
| CH0228.3 | 703010228.3.d0019.ipe025.e4 | 6 | 9 | $1.8 \times 10^{-3}$ |
| CH1244.3 | 703011244.3.d0012.ipe015.3.13 | 2 | 2 | $6.6 \times 10^{-5}$ |
| CH1244.3 | 703011244.3.d0012.ipe015.3.17 | 2 | 2 | $6.7 \times 10^{-5}$ |
| CH1244.3 | 703011244.3.d0012.ipe015.3.6 | 2 | 2 | $1.3 \times 10^{-4}$ |
| CH1244.3 | 703011244.3.d0015.ipe015.3.17 | 2 | 2 | $6.7 \times 10^{-5}$ |
| CH1244.3 | 703011244.3.d0015.ipe015.3.23 | 2 | 2 | $6.6 \times 10^{-5}$ |
| CH1244.3 | 703011244.3.d0015.ipe015.3.30 | 4 | 4 | $4.4 \times 10^{-4}$ |
| CH1244.3 | 703011244.3.d0015.ipe015.3.32 | 4 | 4 | $4.5 \times 10^{-4}$ |
| CH1244.3 | 703011244.3.d0015.ipe015.3.38 | 4 | 4 | $4.5 \times 10^{-4}$ |
| CH1244.3 | 703011244.3.d0015.ipe015.3.4 | 4 | 4 | $5.1 \times 10^{-4}$ |
| CH1244.3 | 703011244.3.d0015.ipe015.3.8 | 4 | 4 | $5.2 \times 10^{-4}$ |

**Supplementary Table 2.** Results from runs of RAPR, RDP4, and the additional tools embedded within RDP4 on 3 sets of 100 randomly generated recombinants, each generated from parental pairs with 0.6%, 1%, and 1.2% diversity respectively.

| **Method** | **Number of unique rec. events detected** | | | **Reference** |
|---|---|---|---|---|
| | **0.6% diversity** | **1% diversity** | **1.2% diversity** | |
| RAPR | 77 | 98 | 92 | Present study |
| RDP4 | -- | 14 | 64 | Martin *et al.* (2015), Vir Evol |
| GeneConv | -- | 15 | 76 | Padidam *et al* . (1999), Virology |
| BootScan | 15 | 15 | 49 | Martin *et al* . (2005), AIDS Res Hum Ret |
| MaxChi | 13 | 41 | 89 | Maynard Smith (1992), J Mol Evol |
| Chimaera | 6 | 28 | 79 | Posada *et al* . (2001), Proc Natl Acad Sci |
| SiScan | -- | 11 | 54 | Gibbs *et al* . (2000), Bioinformatics |
| 3Seq | 8 | 37 | 80 | Boni *et al* . (2007), Genetics |
| PhylPro | -- | -- | -- | Weiller *et al* . (1998), Mol Biol Evol |
| LARD | -- | -- | -- | Holmes *et al* . (1999), Mol Biol Evol |

**Supplementary Table 3.** Estimated rates of coinfection, the percentage of co-infected cells and the half replacement time by recombinants during the infection (5' half genome).

| Subject | Coinfection rate (day$^{-1}$) | % co-infected cells | $T_{1/2}$ (days) |
|---|---|---|---|
| CH0010 | 0.037 (0.021, 0.052) | 1.8 | 42.9 (32.9, 69.0) |
| CH0078 | 0.058 (0.046, 0.069) | 2.8 | 49.9 (43.6, 60.2) |
| CH0200 | 0.05 (0.026, 0.085) | 2.4 | 29.7 (21.9, 46.1) |
| CH0047 | 0.032 (0.014, 0.067) | 1.6 | 50.4 (34.0, 91.0) |
| CH0228 | 0.018 (0.014, 0.035) | 0.9 | 69.0 (45.0, 85.0) |
| CH1754 | 0.096 (0.059, 0.151) | 4.6 | 22.4 (19.3, 27.7) |
| CH0654 | 0.044 (0.033, 0.06) | 2.2 | 23.6 (21.3, 26.3) |
| **Median** | **0.037** | **2.2** | **30.0** |

The rates of coinfection is given as $\gamma\beta I$, and the percentages of co-infected cells during the infection is $F_c = \gamma\beta I/(\gamma\beta I + \gamma\beta T)$ with $\gamma\beta T = 2$ day$^{-1}$. $T_{1/2}$ indicates the predicted time when the frequency of recombinants reaches 50% of the viral population. Coinfection rate was estimated by fitting 3' and 5' data simultaneously using maximum likelihood (see Methods for more detail). In CH0654, all viruses were recombinants at day 84 post infection before the initiation of ART at day 112. Therefore, ART did not affect the analysis of CH0654.

**Supplementary Table 4.** Neutralization potency ($IC_{50}$) of the CH0505 escape variants N279D, V281G, and T234N against autologous antibodies from lineages CH103 and CH235. Data for N279D is from our previous publication (Gao et al., Cell. 2014). VRC01 was included as a control. Response strengths are color coded according to the ranges depicted at the bottom.

| Mutation | Mutation Location | CH235.UCA | CH103.UCA | CH235.IA3 | CH235 bNAb | CH235.9 bNAb | VRC01 |
|---|---|---|---|---|---|---|---|
| TF | | >20 | 2.29 | 6.99 | 0.25 | 0.36 | 0.12 |
| N230D | Δ Glycan 230 | >20 | 2.09 | 6.11 | 0.37 | 0.44 | 0.11 |
| N279D | Loop D | >20 | N/A | >20 | 0.56 | 0.42 | N/A |
| V281G | Loop D | >20 | 0.56 | >20 | 0.47 | 0.19 | N/A |
| N279D + V281G | Loop D | >20 | 0.46 | >20 | 16.19 | 5.78 | N/A |
| T234N | Glycan 234 | >20 | N/A | >20 | 1.89 | 0.63 | 0.87 |

| IC50, ug/ml | <0.1 | 0.1-1 | 1-10 | 10-20 | >20 |
|---|---|---|---|---|---|

**Supplementary Table 5.** Neutralization susceptibility of wild type and recombinants to autologous neutralizing antibodies.

| Virus | | Time point | | | | | | | |
|---|---|---|---|---|---|---|---|---|---|
| | | **w1 (d26)** | **w3 (d40)** | **w4 (d47)** | **w8 (d72)** | **w12 (d100)** | **w16 (d130)** | **w24 (d188)** | **w36 (d275)** |
| **T/F** | **T/F a** | <20 | 45 | 24 | 34 | 378 | 572 | 3,737 | 1,891 |
| | **T/F b** | <20 | 27 | <20 | <20 | 203 | 54 | 4,471 | 1,445 |
| | **T/F c** | <20 | 37 | 22 | 22 | 329 | 374 | 2,831 | 1,977 |
| | **T/F d** | <20 | 39 | 26 | <20 | 66 | 116 | 1,562 | 1,207 |
| | **T/F e** | <20 | <20 | <20 | <20 | 90 | 431 | 492 | 914 |
| **Wild type** | **w8.e5** | <20 | <20 | <20 | <20 | <20 | <20 | <20 | 188 |
| | **w8.e15** | 23 | <20 | <20 | <20 | <20 | <20 | 37 | 26 |
| **Recombinant** | **w8.e1** | <20 | <20 | <20 | <20 | <20 | <20 | <20 | <20 |
| | **w8.e18** | <20 | <20 | <20 | <20 | 20 | <20 | 26 | <20 |
| | **w8.e23** | <20 | <20 | <20 | <20 | <20 | <20 | <20 | <20 |

Pseudovirues made from five T/Fs and five day 72 *env* genes from subject CH0010 were analyzed for their neutralization susceptibility to autologous plasma up to 36 weeks post infection.

**Supplementary Table 6**. Demographic characteristics of individuals infected with multiple and single T/F viruses.

| Subject | Subtype | EDPI. | VL set point | HLA-B allele 1 | HLA-B allele 2 |
|---|---|---|---|---|---|
| **Mulitple T/F virus** | | | | | |
| 703010010 | C | 9 (7, 11) | 181,970 | *1503/61/74/*9503 | *0702/22/23/26/30/39/41/44/46-49N |
| 703010200 | C | 15 (13, 17) | 154,882 | *5301/10 | *3501/04/05/07/09/10/12/13/15-17/20/22/23/29-32/37/40-42/48/51-54/57/64/68 |
| 703010228 | C | 24 (21, 18) | 213,796 | *4201/02 | *1503/61/74/*9503 |
| 703010275 | C | 12 (10, 14) | 87,096 | *44:03/13/26 | *42:01 |
| 703011754 | C | 18 (17, 19) | 645,654 | *08:01/19N/109/115 | *15:10:01 |
| 703011244 | C | 12 (11, 13) | 234,423 | *15:17/196/208/216 | *53:01/23/25/26 |
| 700010654 | B | 27 (24, 29) | ART within 6 mo | *3906 *3906 | *4402/19N |
| 705010078 | C | 11 (7, 14) | 3,548 | *42:01/02 | *81:01 |
| 702010047 | C | 25 (21, 29) | 33,884 | *58:01 | *58:02 |
| **Sinlge T/F virus** | | | | | |
| 705010569 | C | 5 (2, 8) | 417 | *0705/06 | *440301 |
| 706010164 | C | 17 (13, 20) | 575,440 | *440302/07 | *4501/03/07 |
| 705010162 | C | 16 (11, 22) | 114,815 | *44:03/13/26 | *42:02 |
| 705010107 | C | 18 (6, 30) | 447 | *3501/07/15/29/40-42/52-54/57/64 | *3910/16 |
| 703010256 | C | 16 (10, 22) | 18,621 | *1401/04/07 | *5301/10 |
| 703010752 | C | 16 (12, 20) | 83,176 | *14:01 | *53:01 |
| 700010470 | B | 11 (8, 14) | 23,442 | *0801/11/14/15/18/19/22-24/27/29/30N | *4901 |
| 700010649 | B | 42 (30, 53) | 4,266 | *52:01 | *50:02 |
| 703010131 | C | 9 (6, 12) | 22,909 | *4501/03/07 | *1503/47/49/61/74/B*9503 |
| 700010040 | B | 15 (13, 18) | 14,791 | *4402/11/19/22-24/27/33/34/41 | *4001/22/30/33/34/42/43/49/54/55/62/65-67 |
| 703011691 | C | 10 (6, 13) | 147,911 | *45:01/07/13 | *15:10 |
| 703010694 | C | 8 (5, 11) | 77,625 | *42:02 | *15:10 |
| 700010058 | B | 24 (19, 30) | 234 | *1402 | *5701-04/09 |
| 705010185 | C | 17 (13, 21) | 40,738 | *8101/02 | *1503/61/74/*9503 |
| 705010198 | C | 13 (7, 18) | 1,175 | *08:01/08N/15/18/19N | *57:01/03 |
| 705010067 | C | 13 (9, 16) | 40 | *5301/10 | *8101/02 |
| 700010077 | B | 21 (14, 27) | 3,631 | *5301/10 | *5701-04/09 |
| 703010054 | C | 89 (76, 102) | 12,303 | *8101/02 | *0702/07/14/21-23/26/30/33/35/39/41/42/44/46-49N |
| 703010505 | C | 23 (18, 28) | 81,283 | *4202 | *570301 |
| 703010848 | C | 17 (14, 19) | 102,329 | *45:01/03 | *58:01 |
| 703010850 | C | 12 (6, 17) | 15,488 | *18:01/02/03/04/05/06/08/11/17N | *15:10/37 |
| 703011432 | C | 17 (12, 22) | ART within 6 mo | *58:02 | *15:10 |
| 704010042 | C | 32 (18, 45) | 128,825 | *1503/61/74/*9503 | *1801/03-06/08/10-12/15/17/20 |
| 704010083 | C | 20 (11, 30) | 218,776 | *18:01/02/03/04/05/06/08/11/17N | *15:10/37 |
| 704010236 | C | 27 (22, 32) | 134,896 | *0801/19N | *1801/17N |
| 705010110 | C | 39 (31, 47) | 50,119 | *3910 | *5802 |
| 705010264 | C | 30 (24, 37) | 74,131 | *08:01/08N/15/18/19N | *58:02 |
| 703010159 | C | 33 (27, 39) | 8,511 | *8101/02 | *1801/03-06/08/10/11/14/17/20 |

Individuals with neutral HLA alleles, protective HLA alleles, disease susceptive HLA alleles, or both protective and disease susceptive alleles were color-coded in green, red, blue and gray, respectively.
